# Modulating Iron for Metabolic Support of TB Host Defense

**DOI:** 10.3389/fimmu.2018.02296

**Published:** 2018-10-15

**Authors:** James J. Phelan, Sharee A. Basdeo, Simone C. Tazoll, Sadhbh McGivern, Judit R. Saborido, Joseph Keane

**Affiliations:** Department of Clinical Medicine, Trinity Centre for Health Sciences, Trinity Translational Medicine Institute, St. James's Hospital, Dublin, Ireland

**Keywords:** immunometabolism, host-directed therapy, host-directed prevention, iron chelation, tuberculosis, iron metabolism, *Mycobacterium tuberculosis*, HIF1α

## Abstract

Tuberculosis (TB) is the world's biggest infectious disease killer. The increasing prevalence of multidrug-resistant and extensively drug-resistant TB demonstrates that current treatments are inadequate and there is an urgent need for novel therapies. Research is now focused on the development of host-directed therapies (HDTs) which can be used in combination with existing antimicrobials, with a special focus on promoting host defense. Immunometabolic reprogramming is integral to TB host defense, therefore, understanding and supporting the immunometabolic pathways that are altered after infection will be important for the development of new HDTs. Moreover, TB pathophysiology is interconnected with iron metabolism. Iron is essential for the survival of *Mycobacterium tuberculosis (Mtb)*, the bacteria that causes TB disease. *Mtb* struggles to replicate and persist in low iron environments. Iron chelation has therefore been suggested as a HDT. In addition to its direct effects on iron availability, iron chelators modulate immunometabolism through the stabilization of HIF1α. This review examines immunometabolism in the context of *Mtb* and its links to iron metabolism. We suggest that iron chelation, and subsequent stabilization of HIF1α, will have multifaceted effects on immunometabolic function and holds potential to be utilized as a HDT to boost the host immune response to *Mtb* infection.

## Introduction

Tuberculosis (TB) is an infectious disease caused by the bacterium *Mycobacterium tuberculosis* (*Mtb*) and it is estimated that just under one quarter of the global population may be latently infected with *Mtb* (1, 2). TB is responsible for approximately 1.7 million deaths annually (2), making it the biggest infectious cause of death. *Mtb* is an airborne pathogen, spread through aerosols created by coughing. After inhalation and infection with *Mtb*, people attempt to mount an adequate innate immune response to eradicate the bacteria without the help of adaptive immunity (so called “early clearance”) (2, 3). Only 5–10% of infected immunocompetent people progress to TB disease (4, 5). These figures suggest that the vast majority of immunocompetent people exposed to TB produce a robust and adequate immune response to clear the infection asymptomatically. This gives credence to the idea that a defective immune response causes TB disease, and can therefore be therapeutically corrected with host-directed therapies (HDT). We suggest that supporting macrophage metabolism, by manipulating iron availability, has potential as a HDT strategy.

The treatment regimen for TB is based on a combination of up to 4 drugs which have to be taken over protracted periods of time. Inadequate treatment and poor compliance however, have resulted in increasing incidences of multiple and extensively drug resistant TB (MDR and XDR TB respectively) (6). MDR and XDR TB patients have very limited treatment alternatives, thus there is an unmet clinical need for better therapeutic options (6). Basic cellular human research has shed light on many aspects of immunity to *Mtb* and has unveiled immune pathways that may be manipulated therapeutically (7–9). Early in *Mtb* infection, both pro and anti-inflammatory pathways are activated at the same time (7, 10). The aims of some HDT approaches are to manipulate this balance- in other words, to reduce the immune braking system and give the immune accelerator more gas. With this approach, a desired clinical outcome is to reduce the time required to clear the infection. A shorter, better treatment regimen would increase compliance and may reduce incidences of MDR and XDR TB. “Immunometabolism,” (the metabolic changes that underpin the ability of immune cells to mount an immune response) has informed many aspects of immunity to *Mtb*. By understanding and manipulating metabolic pathways, we are seeking to redirect or accelerate the host to yield better clinical outcomes for patients. Iron plays a central role in modulating metabolic pathways (11–13). In this review, we present the evidence suggesting that iron chelation, and its effects on immunometabolism, may be a plausible adjunctive HDT option for TB.

## An overview of immunometabolism

Recent studies on macrophages and metabolic function have linked intermediate metabolism to immune phenotypes (14). It is now considered that immune cell function and cellular energy metabolism are closely coupled and that alterations in metabolic pathways are integral to the immune response, since they strongly influence cell fate and effector functions; these links have been thoroughly reviewed in T cells, macrophages, NK cells, dendritic cells (DCs), and neutrophils (15–20). The metabolic pathways of these cells must be tightly regulated to provide energy and biosynthetic precursors to meet the cells' functional requirements upon activation (21). It has been shown that, upon activation, various immune cells undergo metabolic reprogramming similar to oncogenic cells. For example, in 1927, Otto Warburg observed that neoplastic cells change their metabolism from oxidative phosphorylation (OXPHOS) to aerobic glycolysis (22). This switch to glycolysis also occurs in immune cells that are activated by pro-inflammatory signals, which differ depending on the cell type, and allows them to produce adenosine triphosphate (ATP) more rapidly (albeit less efficiently) and provides the necessary metabolic intermediates needed for cell growth, proliferation and effector mechanisms (23). Glycolytic shifts that take place in activated immune cells are not always classically Warburg (increased glycolysis and reduced OXPHOS) as both glycolysis and OXPHOS can be enhanced simultaneously (24). Increased activity of both glycolysis and OXPHOS is often observed in human immune cells upon activation (24). Furthermore, the metabolic profile of certain immune cells has also been shown to change during different stages of activation. The complex metabolic phenotype defines the function of the immune cell. For example, glycolytic metabolism is associated with classically activated pro-inflammatory macrophages (termed “M1”), effector T cells, cytokine activated NK cells and toll-like receptor (TLR) activated DCs (21, 23–26). This metabolic reprogramming toward increased glycolysis, resulting in increased inflammation, is mediated by two main signaling molecules; mTOR and HIF1α. On the other hand, OXPHOS is generally associated with the phenotype of tissue-resident and alternatively activated macrophages (termed “M2”), long-lived memory T cells, regulatory T cells and mature DCs in their antigen presenting phase (27). It is also worthwhile noting that oxidative metabolism supports immune cell longevity. For example, preserving OXPHOS in activated DCs results in an increased lifespan, and switching cellular metabolism from glycolysis to oxidative metabolism promotes a shift from short-lived M1 macrophages to longer-lived M2 macrophages (28, 29). Human alveolar macrophages (AMs), which are thought to be M2-like, demonstrate greater metabolic plasticity toward glycolytic metabolism upon inhibition of OXPHOS; this is despite a greater reliance of human AMs on OXPHOS at baseline compared to untreated human monocyte-derived macrophages (hMDMs) and IL4 treated hMDMs (30). However, this glycolytic reserve is attenuated in AMs from smokers and in AMs infected with both live attenuated *Mtb* H37Ra and irradiated *Mtb* H37Rv strains (30).

### Monocytes and macrophages

Monocytes are phagocytic and are capable of antigen presentation but are best known as the precursor cells to macrophages and DC. Two key regulators of monocyte metabolism are mTOR and HIF1α, both of which enhance glycolysis (31). The activity and gene expression of these two molecular mediators is enhanced by β-glucan, one of the main components of the fungal cell wall, known to upregulate glycolysis in human monocytes (31). Interestingly, *M.bovis* BCG is also capable of inducing these changes to prime monocytes to respond more rapidly and with heightened activity when challenged by other pathogens, in a process known as innate training (32). The phenomenon of innate training relies fundamentally on changes in glycolytic and glutamine metabolism of monocytes which are crucial for the induction of histone modifications underlying BCG-induced trained immunity (32).

Monocytes extravasate from the blood into the tissue where they differentiate into macrophages or DCs (33, 34). Macrophages can have pro-inflammatory or pro-resolution phenotypes depending on the cytokine milieu they experience and pathogen- or damage- associated molecular patterns (PAMP/DAMP) signals they receive *in situ* (35). These are broadly classified as classically activated or M1-type macrophages and alternatively activated or M2-type macrophages (36). M1- and M2-type macrophages differ in terms of function and in the metabolic pathways they utilize; in fact, differences in metabolic function direct their differentiation and phenotype (36, 37). M1 macrophages confer protection against bacterial infection via a pro-inflammatory response, involving several cytokines, nitrogen species and pro-inflammatory reactive oxygen species (ROS) (38). Murine macrophages, activated with LPS, have been demonstrated to rely on glycolysis to produce ATP, which is primarily mediated by HIF1α (39). Glycolysis in LPS-IFNγ stimulated murine bone marrow-derived macrophages (BMDMs) is also directed toward the pentose phosphate pathway (PPP) and the malate-aspartate shuttle to support NADPH synthesis, essential for ROS production (40). M2 macrophages, involved in tissue homeostasis and wound healing, mediate Th_2_ cell immunity to parasitic infections, which are usually chronic and therefore energy demanding (41). In keeping with the longevity of their role, M2 macrophages independently stimulated with IL4, signal transducer and activator of transcription 6 (STAT6) and PPARγ-coactivator-1β (PGC-1β) engage an anti-inflammatory phenotype and rely on fatty acid oxidation (FAO) to generate ATP (42).

In murine BMDMs, LPS stimulation results in increased glycolysis (39) and a break in the tricarboxylic acid (TCA) cycle at two points; one at citrate and another at succinate. Succinate drives the production of IL1β, mediated by HIF1α (39) whereas citrate accumulation leads to the production of itaconate, a potent inhibitor of isocitrate lyase, which is necessary for *Mtb* persistence (43). Itaconate has anti-inflammatory and anti-oxidant properties, mediated by NRF2 signaling (44), as well as being directly able to effect *Mtb* growth (45). It has also long been known that lipid metabolism is significantly altered during infection and inflammation (46–48). Increased lipid uptake leads to foam cell formation, or foamy macrophages, which is characteristic of certain diseases such as atherosclerosis and TB (49, 50). Many experimental models also utilize LPS as a stimulus, however, the use of LPS can have its limitations (51).

Macrophages, especially alveolar macrophages, are crucial in *Mtb* infection as they are probably the first cell to encounter *Mtb* and become infected. Macrophages are both critical to the eradication of the infection but are also culpable of harboring *Mtb* thus, propagating the infection. We hypothesize that these divergent processes are hinged on the metabolic potential of the macrophage. Macrophage metabolism during *Mtb* infection will be further explored in the section below entitled “*Metabolic alterations within immune cells during Mtb infection.”*

### Neutrophils

Neutrophils play vital roles during infection, since they contribute directly to the elimination of pathogens via phagocytosis, the release of antimicrobial molecules such as hydrogen peroxide and netosis, a process whereby activated neutrophils form net-like structures to trap the pathogens (52). These key features of neutrophil activity rely on neutrophil phagocytosis and the switch toward aerobic glycolysis (52, 53). Interestingly, neither glucose nor glutamine are fully oxidized to produce ATP in these cells, indicating that glycolysis may be supporting alternative metabolic pathways through the production of intermediates to generate antimicrobial molecules (53). Indeed, glucose is metabolized to fuel the PPP to generate NADPH in healthy human neutrophils stimulated with PMA and amyloid fibrils (54). Moreover, degradation of glutamine to malate via the TCA cycle and the malate-aspartate shuttle, contributes to the generation of NADPH. NADPH production is essential for the microbicidal cytosolic NADPH oxidase (NOX) system, required for netosis and for production of antimicrobial molecules (55, 56). HIF1α also mediates glycolytic metabolism in murine neutrophils as conditional knockout of HIF1α drastically reduced ATP levels resulting in impaired bacterial killing (57). Neutrophils are implicated in early TB host defense but also contribute to tissue pathology, especially later on in disease. The metabolic profile of neutrophils during *Mtb* infection is not yet known, however; targeting metabolic pathways in these cells may help to fine-tune the immune response to promote clearance or inhibit tissue damage.

### Natural killer cells

Natural killer (NK) cells, activated by IL2, IL12, IL15 or combinations thereof, have increased glucose metabolism through aerobic glycolysis, which is necessary to meet the requirements for rapid growth and proliferation (58–60). Specifically, healthy human NK cells are classified into two distinct subsets based on their levels of CD56 receptor; CD56^DIM^ cells are considered more cytotoxic whereas CD56^HI^ cells are potent producers of IFNγ (24). Flow cytometric analysis showed that CD56^HI^ cells express higher levels of the glucose transporter GLUT1 and exhibit higher glycolytic metabolism than CD56^DIM^ cells. Although OXPHOS supports both CD56 cell subtypes, limiting glycolysis in CD56^HI^ cells significantly impairs the production of IFNγ, a pro-inflammatory cytokine also central to host defense during *Mtb* infection (24). Both subpopulations of NK cells respond to *Mtb* and can directly kill *Mtb* infected phagocytes through the production of perforin, granzyme and the ligation of death receptors (24, 61). Indirectly, healthy human NK cells promote host defense in *Mtb* H37Ra-infected T cells by producing IFNγ and inducing CD8^+^ T cell responses (62). Interestingly, memory-like antigen-specific CD45RO^+^ NK cells, isolated from the pleural fluid from patients with tuberculosis, exhibit features of innate memory to *Mtb* antigens and may participate in the recall immune response to *Mtb* infection by producing IL22 (63, 64). This is similar to BCG-induced innate training observed in human and murine monocytes *in vitro*, which are dependent on glycolytic and glutamine metabolism (32). The metabolic changes in NK cells during *Mtb* infection are not yet characterized but are likely to be integral to its host defense mechanisms.

### Dendritic cells

In DCs, similar to other immune cells, cell function is coupled to immunometabolism with the aim of meeting the bioenergetic and biosynthetic requirements for successful TLR induced activation and function (25, 65). TLR-activated DCs stimulated with LPS, heat killed *Propionibacterium acnes* or CpG, rely on aerobic glycolysis to generate ATP (65, 66). This switch to glycolysis is primarily regulated by HIF1α and the PI3K/Akt pathway (65, 67). Additionally, in real-time extracellular metabolic flux assays, the change to glycolytic metabolism has been shown to enhance nitric oxide (NO) synthesis via the enzyme nitric oxide synthase 2 (NOS2), which inhibits OXPHOS in some populations of LPS stimulated human DCs (66). Therefore, glucose plays two roles in DC activation post TLR stimulation; in the early stages of activation, glucose provides the metabolic intermediates needed for DC maturation. However, in subsequent stages, NO production inhibits OXPHOS, making glycolysis necessary to synthesize ATP and support cell survival (66). Linking DC metabolism and function to adaptive immunity, glycolysis has also been shown to repress the pro-inflammatory output of BMDM-derived LPS-stimulated murine DCs and limit DC-induced T cell responses (27). Therefore, the lifecycle of the DC is marked by differences in metabolism intrinsic to the function of the DC at that stage. DCs play a crucial role in propagating T cell responses during *Mtb* infection, however, their metabolic phenotype is understudied. For example, one study characterized the cooperation between *Mtb*-infected human CD1c^+^ DCs and plasmacytoid DCs which favors the stimulation of CD4^+^ T cells, and another study has identified the rapid induction of glycolysis as an integral component of TLR signaling that is essential for the anabolic demands of the activation and function of murine DCs (25, 68). We hypothesize that metabolism may underpin DC function during *Mtb* infection, based on such observations.

### T cells

Resting T cells rely primarily on OXPHOS, however, once activated by the T cell receptor and costimulatory molecule ligation, T cell subsets undergo a distinct metabolic reprogramming (69). In the early stages of inflammation, cytokines direct the differentiation of naïve CD4^+^ T cells into effector (T_eff_: Th_1_, Th_2_ or Th_17_) or inducible regulatory T cell (T_reg_) subsets (70–76). Effector T cell subsets show an increase in glycolytic metabolism following activation, namely Th_17_ cells, Th_1_ and Th_2_ cells (21, 23, 77). Consistent with the different functions of these subsets, T_eff_ and T_reg_ cells utilize distinct metabolic programmes. Murine T_eff_ cells depend on aerobic glycolysis to enable the rapid growth and proliferation essential for clonal expansion, migration and effector functions (21). Alternatively, T_reg_ cells have less of the glucose transporter GLUT1 on the surface and rely on lipid oxidation and OXPHOS to generate ATP (21). Extracelular flux and flow cytometry analyses demonstrate that murine CD8^+^ memory T cells primarily rely on lipid oxidation, the TCA cycle and OXPHOS, utilizing extracellular glucose to synthesize lipids rather than using extracellular fatty acids directly (78, 79) whereas activated effector CD8^+^ T cells shift their metabolism toward glycolysis. Myc, HIF1α, estrogen related receptor-α and mTOR are some of the molecular mediators critical to driving these alterations in T-cells. Myc upregulates various genes involved in glucose and glutamine metabolism in the initial stages of T cell activation in primary murine cells (69). Similarly, the mTOR pathway promotes glucose metabolism in human T_eff_ cells while inhibiting T_reg_ generation (80). Moreover, in an mTOR dependent manner, HIF1α is a critical regulator of the Th_17_ and T_reg_ axis through the modulation of glycolytic metabolism in murine cells (77). In recent years, it has emerged that, in certain settings of inflammation, significant plasticity occurs between Th_1_, Th_17_ and T_reg_ cell lineages (81, 82). Given their differential metabolic states, it is plausible that metabolic reprogramming underpins and directs the plasticity of these cells. The metabolic status of CD3^+^ T cells was recently examined in an *in vivo* mouse model of *Mtb* infection where the authors showed that the T cell compartment in granulomatous regions of the lungs have increased transcripts encoding glucose transporters, glycolytic enzymes and enzymes of the pentose phosphate pathway (83). These alterations, and further increases in the expression of hexokinase-3 and lactate dehydrogenase A in co-localization analyses, may be indicative of increased glycolytic metabolism (83). Even though further studies are warranted to explore this link, T cell exhaustion in *Mtb* infection is postulated to be linked to metabolism, especially in the oxygen-deprived environment of the granuloma. Modulating T cell metabolism may therefore be beneficial in promoting a specific T cell response with the capacity to support *Mtb* clearance, particularly during the early stages of infection.

## Metabolic alterations within immune cells during *Mtb* infection

### Glycolysis and oxidative phosphorylation

Upon *Mtb* infection, the immune system aims to contain and eradicate the pathogen. However, infected cells such as macrophages, are sometimes unable to eliminate *Mtb*, thus favoring the formation of granulomas to contain the infection (84). As immune cells in these granulomatous structures need to be functionally committed to controlling the infection, it is crucial that their metabolic activity meets the bioenergetic and biosynthetic requirements needed to efficiently clear or contain the pathogenic burden. Infection of hMDMs, AMs and murine BMDMs with the irradiated *Mtb* H37Rv strain of *Mtb* is associated with increased extracellular lactate levels, indicative of an increase in glycolysis (7). Increased extracellular lactate levels were also enhanced in all macrophage cell types when infected with the live attenuated *Mtb* H37Ra strain and the live *Mtb* H37Rv strain (7). In addition, transcriptomic analysis of murine lungs infected with *Mtb* has revealed that during infection, genes involved in glucose metabolism are upregulated whilst genes that encode enzymes from the TCA cycle and OXPHOS are downregulated, indicating the occurrence of a Warburg effect (83). This switch is further evidenced in a NMR-based metabolomic profiling study showing increased concentrations of lactate in granulomas from *Mtb*-H37Rv-infected C57BL/6 mice (85). Moreover, the shift toward aerobic glycolysis during *Mtb* infection is linked to the ability of human macrophages to produce mature IL1β, subsequently demonstrated to be essential for bacteriocidal activity against *Mtb* when glycolysis was blocked with the glycolytic inhibitor 2-deoxyglucose (2-DG) (7).

Depending on their ontogeny, tissue resident macrophages and infiltrating macrophages have distinct roles, phenotypes and display differential metabolic profiles. In a mouse model of *Mtb* Erdman infection, *Mtb* has been shown to trigger the accumulation of interstitial macrophages (IMs) (Ly6C^high^, CX_3_CR1^+^, and CD11b^high^) derived from blood monocytes that are phenotypically distinct from tissue resident AMs (Siglec F^+^ and CD11c^high^) (86). In this murine model, IMs were found to be more glycolytically active than AMs, with the latter cells relying more on FAO and fatty acid uptake (86). Interestingly, depletion of AMs reduced bacterial burden whereas deletion of IMs increased bacterial burden suggesting that AMs are permissive to *Mtb* (86). Furthermore, inhibition of glycolysis by 2-DG decreased the number of IMs and concomitantly increased bacterial burden thereby coupling metabolism with cellular function.

Human AMs exhibit significantly higher extracellular lactate levels, indicative of increased glycolysis, upon infection with the *Mtb* H37Ra and *Mtb* H37Rv strains (7). We have recently found that human AMs are also just as energetically responsive as hMDMs, which describes the ability of a cell to respond metabolically when stressed (30). For example, upon oligomycin-induced inhibition of OXPHOS in human AMs and hMDMs, AMs compensate by increasing glycolytic metabolism just as effectively as hMDMs (30). Others have also shown that human AMs contain *Mtb* better than monocytes (87). Furthermore, blocking glycolysis using the alternative carbon source, galactose, resulted in increased bacterial load in the human AMs, suggesting that this metabolic shift is required in AMs to allow them to exert bacillary killing (7). 2-DG reduced IL1β production in murine BMDMs, hMDM and human AMs, further supporting the idea that the switch to glycolysis is essential for optimal IL1β production, crucial to the control of bacillary replication (7). Production of IL1β is regulated by HIF1α, which is stabilized upon inhibition of the prolyl hydroxylase domain (PHD) proteins (88). As HIF1α lies at the crux of the glycolytic switch, HDTs that target PHD proteins and stabilize HIF1α may effectively boost glycolytic metabolism thereby supporting defense mechanisms within infected host immune cells.

### Amino acid metabolism

In addition to this glycolytic switch in energy metabolism, amino acid availability in the granuloma plays a key role in *Mtb* infection in human and murine studies (89–92). Amino acids are not only essential for cytokine and chemokine synthesis, but they play a role in the production of anti-microbial agents. More specifically, three amino acids, L-arginine, L-tryptophan and L-glutamine are key regulators of immunometabolism in TB (93–97). During TB infection, L-arginine is implicated in several immune cell effector functions, including the production of NO, and may therefore be important in the outcome of the infection (95). It has also been demonstrated that M2 macrophages express arginase-1 (Arg1), an enzyme that hydrolyses L-arginine to ornithine and urea (90). When macrophages express both Arg1 and inducible nitric oxide synthase (iNOS), NO synthesis is limited as both enzymes consume L-arginine. Abrogation of macrophage Arg1 exacerbates *Mtb* H37Rv growth and pathology in murine TB lung granulomas (89). Moreover, Arg1 plays an important role in L-arginine withdrawal from T cells within the same granuloma, leading to T cell inhibition (89). Hence these two functions of Arg1 may contribute in limiting the host cell's response to TB infection and protect the host from immune-mediated damage.

In response to *Mtb* infection, macrophages strongly upregulate the expression of indoleamine 2,3-dioxygenase enzymes (IDO1, IDO2, and TDO), that convert L-tryptophan into L-kynurenine. L-tryptophan catabolism has been demonstrated in transcriptomic and flow cytometry analyses to inhibit murine T_eff_ cell function and induce CD25^+^Foxp3^+^ T_reg_ subsets, reducing immune activity, limiting tissue damage and favoring pathogen survival (98). Furthermore, IDO-expressing DCs are essential for maintaining granulomas, which contain *Listeria monocytogenes* and enable mycobacterial survival (99). More recently, cerebral tryptophan metabolism has also been shown to be important for the outcome of tuberculous meningitis, where low cerebrospinal fluid tryptophan concentrations strongly predicted patient survival (100). Hence, modulating L-tryptophan metabolism could be used as a potential HDT strategy.

Glutamine is synthesized in a reaction catalyzed by the enzyme glutamine synthetase from L-glutamate, ammonia and ATP (96). Given the importance of this enzyme in nitrogen metabolism, it is believed to influence *Mtb* pathogenesis by altering ammonia levels within infected cells and thus may contribute to *Mtb*-mediated inhibition of phagosome-lysosome fusion and acidification (101). Conversely, L-glutamate exhibits potential to be utilized in the production of additional succinate, a TCA cycle intermediate now known to play an important role in the production of IL1β in LPS-stimulated murine BMDMs, mediated by the reverse electron transport process, in a ROS-HIF1α dependent manner (39, 102).

### Fatty acid metabolism

Fatty acid metabolism is another key aspect of TB that effects both *Mtb* and the infected host. Lung resident AMs are influenced by ongoing exposure to and uptake of surfactant, a lipid-protein complex that lowers surface tension and aids inhalation. When hMDMs are treated with surfactant *in vitro*, the growth of *Mtb* H37Rv is increased due to increased intracellular levels of the lipid, which the bacteria can use as a carbon source (103). Lipids serve as a key nutrient and energy source, but they also participate in regulating other immune responses. For instance, triacylglycerols (TAGs) can reduce *Mtb* H37Rv growth and antibiotic sensitivity, and the equilibrium between fatty acid synthesis and degradation may alter redox homeostasis in the cytosol (104, 105). Several studies have also demonstrated that *Mtb* utilizes cholesterol and fatty acids as essential nutrients during infection and *Mtb* preferentially metabolizes host lipids, although it can utilize a variety of nutrients to obtain energy (106, 107). Flow cytometry and co-localization analyses show that intra-phagosomal lipolysis is also markedly reduced in conjunction with the retention of host lipids further providing a potential source of nutrients for hMDMs and murine BMDMs infected with the *Mtb* CDC1551 strain (108).

The ability of *Mtb* to perturb fatty acid metabolism during infection results in the formation of foamy macrophages (106, 107). This is thought to be mediated by TLR2 signaling and increased PPARγ in human macrophages infected with *Mtb* H37Rv, killed *Mtb* H37Rv, *M. smegmatis* and *M.bovis* resulting in lipid droplet accumulation (109). Low density lipoproteins containing cholesterol, TAGs and phospholipids, are sequestered within macrophages. Whilst TAGs and phospholipids are metabolized, the cholesterol is then either exported through ATP-binding cassette transporters, or esterified and accumulates as droplets, which leads to the formation of foamy macrophages (110). Traditionally, the function of foamy macrophages was thought to be restricted to lipid storage, however, it has now been shown that they may be essential for mycobacterial persistence and reactivation (111–113). For example, the bacterial glyoxylate shunt enzymes isocitrate lyase 1 and 2 are required for bacterial growth and virulence of *Mtb* Erdman-infected hMDMs and murine BMDMs *in-vitro*, and in an *in-vivo* murine model (111). *Mtb* H37Rv-infected murine BMDMs also require the utilization of cholesterol for survival during prolonged infection (112). Moreover, the accumulation of lipids has a significant impact on the metabolic pathways within *Mtb*, as the mycobacteria must produce more lipolytic enzymes to degrade these host lipids, especially cholesterol (107). Cholesterol degradation generates propionyl-CoA, which is a potential source of toxic metabolites that could compromise *Mtb* survival. Hence *Mtb* metabolizes this precursor toward different metabolites, by balancing acetyl-CoA and propionyl-CoA concentrations, some of which can be used to build the lipid elements of the cell wall, which not only support the structure, but are also important virulence factors (114). Thus the ability of *Mtb* to utilize host-derived lipids effectively is key to its success as a pathogen. Beyond providing a nutrient source and building blocks for bacterial growth, this accumulation of lipids in human cells can also block host autophagy and lysosome acidification, two other essential mechanisms for the control of *Mtb* (115). Others suggest that the accumulation of lipid droplets is the result of macrophage activation (not *Mtb*-induced perturbations) as it is dependent on IFNγ and HIF1α mediated glycolytic reprogramming in murine BMDMs (116). Interestingly, *Mtb* Erdman is able to acquire host lipids in the absence of lipid droplets, but not in the presence of IFNγ-induced host derived lipid droplets, thereby uncoupling macrophage lipid formation from bacterial acquisition of host lipids (116). These IFNγ-induced lipids, which require HIF1α for their synthesis, support the production of host protective eicosanoids including LXB4 and PGE2 (116). In addition, it has been demonstrated that lipid droplet formation is necessary for the production of host protective eicosanoids. Taken together, these changes in FA metabolism during *Mtb* infection suggest that targeting FA metabolism could result in the development of new and improved HDTs.

## HIF1α is a key regulator of immunometabolism during *Mtb* infection

HIF1α is central to reprogramming metabolism toward utilizing aerobic glycolysis; a process that functions as a key gate keeper in immune cell activation. As HIF1α is central to various preneoplastic and neoplastic diseases, it is not surprising therefore that HIF1α has been identified as a crucial molecular mediator during *Mtb* infection in humans and mice (8, 116–118). HIF1α is required for the production of NO, IL1β and prostaglandin E2 (PGE2) as demonstrated by murine HIF1α knockout macrophages which exhibit impaired production of these key cytokines in response to *Mtb* infection (8). HIF1α is also self-sustaining as stabilized HIF1α expression promotes glycolysis during *Mtb* infection, and this enhanced aerobic glycolysis promotes further stabilization of HIF1α (8). NO modulates macrophage responses to *Mtb* infection in murine BMDMs, through transcriptional and protein activation of HIF1α (119). HIF1α and iNOS are linked by a positive feedback loop that elicits further macrophage activation and regulate aerobic glycolysis (119). Specifically, *Nos2*^−/−^ and *HIF1*α^−/−^ knockout results in significant transcriptional defects in various glycolytic genes including *GLUT1, LDHA*, and *PFKFB3* by RNAseq analysis (119). This results in significant reductions in extracellular glucose consumption in these BMDMs (119). Moreover, when murine BMDMs are activated with LPS, this results in an increase in the TCA metabolite succinate (39). Succinate is implicated in various different cellular mechanisms, such as inducing TLR synergy, participating in important post-translational modifications and in propagating further enhancement of glycolysis (39). Accumulation of succinate promotes the stabilization of HIF1α resulting in increased IL1β production from murine BMDMs (39).

HIF1α is also capable of binding to the promoter region of *pfkfb3* (120). The gene *pfkfb3* encodes an isoform of 6-phosphofructo-2-kinase/fructose-2,6-biphosphatase (PFKFB3). This enzyme regulates the production of fructose-2,6-biphosphate, a glycolysis intermediate which activates 6-phosphofructo-1-kinase, and this increases glucose uptake (121). Several studies have shown that *pfkfb3* levels increase after infection with *Mtb* in mouse, rabbit and human lungs (83, 122, 123). This upregulation is thought to be strongly dependent on HIF1α (83).

HIF1α is also crucial for the IFNγ-dependent control of *Mtb* in an *in vitro* and *in vivo* study of mice, as it mediates the metabolic switch to glycolysis in *Mtb* Erdman-infected BMDMs (8). IFNγ promotes M1-type macrophage polarization, cytokine production and synthesis of microbicidal mediators such as NO during infection with *Mtb* Erdman (124). Furthermore, HIF1α acts a positive feedback mediator during this process and acts to sustain the role of IFNγ in macrophage activation, helping to control and restrain the infection. This sustained metabolic transition to aerobic glycolysis is thought to be vital for IFNγ to successfully control the immune response to *Mtb* (8).

Mechanistically, the stabilization and subsequent activity of HIF1α is tightly regulated by a family of PHD proteins which continually target HIF1α for proteasomal degradation during homeostasis (125). If HIF1α is degraded, this turns off the regulation of all metabolic pathways where HIF1α is involved, such as the metabolic switch toward aerobic glycolysis. PHD enzymes require oxygen and α-ketoglutarate (αKG) as co-substrates, in addition to iron and ascorbate (126), hence the regulation of HIF1α by PHD enzymes is tightly associated with iron availability. Therefore, we suggest that the therapeutic chelation of iron may serve to disable PHD activity and promote the stabilization of HIF1α which may in turn promote bacterial clearance during *Mtb* infection through enhanced immunometabolism and increased effector functions.

## Immunometabolism and iron are intrinsically linked.

### Controlling physiological levels of iron

**Figure 1:**
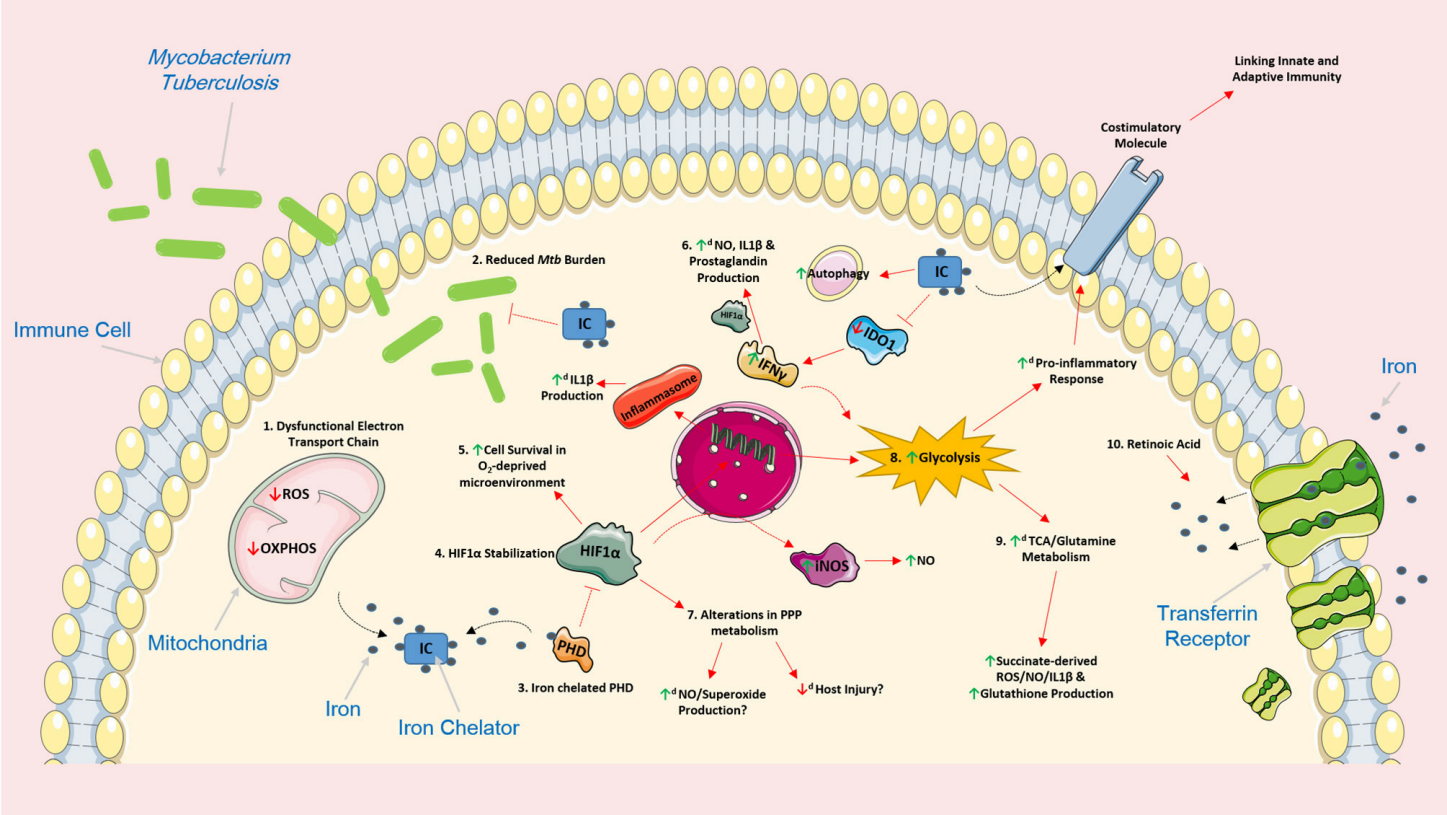
Iron chelation encompasses the ability to support the host response by modulating cellular function and metabolism in various *Mtb-*infected immune cells. The use of iron chelators could potentially regulate a host of intracellular networks and support infected host cells by influencing several cellular processes. 1. Iron chelation results in a dysfunctional electron transport chain (ETC) as the ETC relies heavily on iron for optimal cellular function. A dysfunctional ETC could result in decreased production of ROS and a reduced capacity to undergo oxidative phosphorylation potentially promoting glycolysis. 2. Iron chelators have also been previously shown to have direct and indirect bacteriostatic and bactericidal effects on *Mtb*. 3. Iron chelation directly inhibits prolyl hydroxylase domain (PHD) proteins, proteins that normally function to silence HIF1α, by chelating one of its primary cofactors, iron. 4. Inhibition of the PHD proteins, through iron chelation, leads to the stabilization of HIF1α which can have various effects on cell function. 5. HIF1α plays an important role in promoting cellular survival in an oxygen-deprived microenvironment such as hypoxia. 6. HIF1α can also induce the production of IL1β, an important pro-inflammatory cytokine that helps control *Mtb* replication, by directly binding to the promotor of pro-IL1β. 6. IFNγ can boost production of nitric oxide (NO), IL1β and prostaglandin (e.g., PGE2) production, via HIF1α. Iron chelation can also inhibit IDO1, a key enzyme in tryptophan metabolism, and promote additional IFNy production. Moreover, iron has been shown to increase the autophagic process. 7. The effect of HIF1α and iron chelators on pentose phosphate pathway (PPP) metabolism remains unclear, however, such alterations in this pathway could be beneficial. Increased NO and superoxide production can help kill unwanted infectious agents, and as the PPP is linked to NADPH and ROS production, decreased activity of this pathway could potentially reduce host injury and increase flux through glycolysis. 8. Iron chelators also encompass the ability to significantly boost glycolysis; such boosts in glycolysis are linked to the production of a host of pro-inflammatory mediators and the expression of various costimulatory molecules which could also link innate and adaptive immunity during *Mtb* infection. 9. By supporting glycolysis, iron chelators could also simultaneously enhance the activity of both the TCA cycle and glutamine metabolism which are intrinsically linked to the production of succinate, ROS, NO, IL1β, and glutathione. 10. The effect of iron chelation on these cellular processes could also be further augmented when administered in combination with other host directed therapies during *Mtb* infection. For example, retinoic acid can promote internalization of the transferrin receptor and further limit intracellular iron stores thereby reinforcing the effect of iron chelation. Image produced with the aid of Servier Medical Art software (see copyright license at https://smart.servier.com).

The activity of HIF1α can be significantly influenced by iron availability (127). Iron is crucially important for many physiological processes. The most common forms of iron in the human body are ferrous (Fe^2+^) and ferric (Fe^3+^) iron; under physiological O_2_ concentrations, the most stable form is Fe^3+^ (128). Body stores of iron are usually assessed by transferrin saturation levels (normal range 20–30%), serum ferritin levels (normal range above 150 ng/mL), serum iron levels (normal range 60–110 ng/mL) and total iron binding capacity (normal range 240–300 ng/mL) but these levels can also vary between sexes (129, 130). Recent data examining iron distribution in freshly resected lungs of TB patients and healthy controls showed that the lungs of *Mtb-*infected patients contain more iron (54.7 ± 6.9 μg/g tissue) than healthy controls (19.4 ± 2.9 μg/g tissue) (131). Dietary iron is absorbed from the duodenum and upper jejunum (132). Here, the divalent metal transporter-1 (DMT1) transports Fe^2+^ and H^+^ into the cell. In the cell, Fe^2+^ is stored within ferritin heavy and light chains, the primary iron storage protein, or it is transported into the blood when required. The iron exporter, ferroportin-1 enables the movement of iron out of cells (132). In the blood, oxidized Fe^3+^ binds to transferrin and can be transported in this state until it reaches its target cell and binds to transferrin receptor-1 on the cell's surface (133). The transferrin-bound iron-transferrin receptor-1 (TBI-TfR1) complex is taken into the cell through the process of endocytosis, ultimately resulting in the release of Fe^3+^ and recycling of transferrin and its receptor (133). Next, iron enters the mitochondria where it is a fundamental component in the synthesis of heme and iron-sulfur cluster-containing proteins which have a central function in the operation of the electron transport chain (134, 135). Thus iron metabolism plays a central role in regulating mitochondrial metabolism pathways. On a cellular level, iron levels are regulated by the iron regulatory protein (IRP)/ iron response element (IRE) system which controls the expression of several proteins essential for iron homeostasis, including DMT1 (136). The expression of hepcidin, a key regulator of the entry of intracellular iron stores into the circulation, is dependent on systemic iron levels (137). Hepcidin binds to ferroportin-1, inhibits it by promoting its internalization and degradation, thus negating iron export out of the cell subsequently lowering the amount of iron entering the circulation (136, 137). Iron is also responsible for oxygen transport and therefore regulates the bioavailability of oxygen in the cell (12). The presence of oxygen promotes the TCA cycle and OXPHOS (12). Conversely, when iron levels are low, there is less oxygen transport, and cells have a reduced oxygen supply. This can result in a decrease in mitochondrial metabolism, and an upregulation in anaerobic glycolysis to compensate for the reduction in ATP generated (12). Thus iron can be intrinsically linked to cellular metabolism and cell function in various ways, as Figure 1 illustrates.

### Exploiting iron chelators for therapeutic gain

The therapeutic utilization of iron chelators has been widely reviewed (138–140). Iron-related pathologies occur when there is an excessive or insufficient level of iron (140). For example, iron overload typically arises from hereditary haemochromatosis. Iron chelation therapy is administered to prevent this, or reverse complications that may have already developed (138, 139). FDA-approved iron chelators, such as desferrioxamine (DFX), bind to free reactive iron in circulation (139). This complex is excreted from the body, thus reducing iron levels (139). Iron chelation therapy currently has many alternative applications. For example, deferiprone has been used in murine models of chronic obstructive pulmonary disease to transfer iron out of the mitochondria, and has been shown to ameliorate cigarette smoke-induced bronchitis and emphysema (141). Iron chelation also holds promise for the treatment of *Plasmodium falciparum*, which causes malaria, as use of deferiprone has been shown to reduce recovery time and increase clearance of the infection (142). DFX has also been shown to reduce the replication of HIV (143). Moreover, the iron chelators deferiprone, Apo6619, and VK28 have all been shown to possess antibacterial qualities against *Staphylococcus aureus* and *Escherichia coli* (144). Unsurprisingly, in addition to their chelating ability, specific iron chelators probably encompass additional properties that functionally set them apart from other iron chelators, elements of which are yet to be determined. For example, deferiprone is known to prevent the growth of coagulase-negative staphylococci but DFX promotes its growth (145). Therefore, the novel approach to treating bacterial infections with iron chelators could prove beneficial in TB, and may even hold promise against multi-drug resistant strains of the bacteria.

### Ironing out *mycobacterium tuberculosis*

*Mtb* requires iron for survival and competes with the host for the same iron pool. To compete for iron, *Mtb* releases siderophores, namely exochelins, which have a high-affinity for iron and can remove it from the host's iron-binding molecules (146). Exochelins subsequently transfer iron to mycobactins in the cell wall of *Mtb* (146). Once iron is accessed, it is strictly controlled for the same reasons as seen in host cells; to maintain homeostatic levels, while preventing toxic accumulations. *Mtb* has readily evolved to utilize iron and controls iron uptake at a transcriptional level (147). The mycobacterial iron-dependent regulator (IdeR) is crucial to the maintenance of iron homeostasis in *Mtb* as experimentally-induced lack of IdeR results in an accumulation of iron, leading to oxidative damage and subsequent death of the mycobacterium, thus highlighting the importance of exochelins and IdeR as *Mtb*-survival mechanisms (147).

Macrophages play a key role in recycling iron. Excessive levels of iron have been documented in macrophages and hepatocytes from populations in Sub-Saharan Africa (148, 149). These populations are linked with a 3.5-fold increase in the probability of developing pulmonary TB (149). Excessive levels of iron are also seen in the macrophages of HIV patients, due to chronic blood transfusions or inflammation (150). Additionally, smoking increases the risk of developing active TB; this may be due in part to increased iron loading AMs. In fact, iron levels in AMs are over 3.2-fold higher in asymptomatic smokers and up to 5.6-fold higher in symptomatic smokers compared with nonsmokers; this could rise to 5.4-fold and 9.2-fold respectively when experimental variation is taken into account (151). It is also well known that iron starvation greatly affects *Mtb*'s ability to proliferate. *Mtb* also adapts to low iron levels by upregulating the expression of various factors such as the ESX-3 secretion system, which facilitates its survival (152). Although there is no direct proof of cause or effect, clinicopathological analysis of iron distribution within human lung tissue shows that *Mtb* severely disrupts iron homeostasis in distinct microanatomic locations of the human lung thus potentially contributing to lung immunopathology (131).

Targeting the hepcidin-ferroportin axis may have clinical utility and could be exploited as a means to alter intracellular iron levels. TLR agonists, except TLR2, were shown to polarize murine BMDMs into pro-inflammatory macrophages and upregulate hepcidin transcript levels (153). By measuring a combination of transcript and protein levels of hepcidin and ferroportin, another study showed that differential TLR signaling can induce intracellular iron sequestration in THP-1 human macrophages (154). Specifically, agonists to TLR1/2, TLR2, and TLR6 significantly reduced transcript levels of ferroportin in THP-1 cells without affecting transcript levels of hepcidin (154). Conversely, TLR4, TLR7, and TLR8 agonists significantly induced both transcript and protein levels of hepcidin without affecting transcript levels of ferroportin (154). More significantly however, both alterations in hepcidin and ferroportin resulted in iron sequestration, suggesting that targeting these may be therapeutically beneficial. For example, by targeting hepcidin, this may reduce intracellular iron sequestration potentially affecting the growth of siderophilic bacteria, such as *Mtb*, while enhancing metabolism though HIF1α stabilization. Indeed, the same study shows that hMDMs infected with the *Mtb* Erdman strain induce high protein levels of hepcidin (154). Interestingly, heparin treatment has recently been shown to reduce hepcidin transcript and protein levels in THP-1 human macrophages infected with BCG and *Mtb* Erdman (155). Moreover, heparin treated macrophages exhibited higher ferroportin transcript and protein levels, promoting iron export and decreasing iron availability to intracellular bacilli. These infected heparin-treated cells also induce increased protein levels of IL1β further rendering hepcidin and ferroportin as attractive therapeutic targets (155).

Macrophage membrane-bound compartments, such as phagosomes and lysosomes, contain the natural resistance-associated macrophage protein-1 (NRAMP1). Murine studies using *M. avium-*infected BMDMs have shown that NRAMP1 acts to protect the host (156, 157). Moreover, several 3′UTR polymorphisms in this protein in humans have been shown to increase susceptibility to TB in specific populations (158–160). Mechanistically, NRAMP1 creates Fe^2+^ efflux from the cell, and TB patients with these NRAMP1 polymorphisms are deprived of this protective method which would normally restrict *Mtb* growth (156, 158, 161). The use of iron chelators on individuals with NRAMP1 polymorphisms could potentially provide the protection that they require. It is also likely that current anti-TB drugs, and other HDTs, administered in combination with iron chelators may result in better clinical outcomes. For example, retinoic acid has re-emerged as a potential HDT as it has been shown to promote cell-mediated clearance of *Mtb* H37Ra-infected BMDMs, hMDMs and human AMs (162). Moreover, retinoic acid has been reported to significantly reduce transferrin receptors on the membrane of macrophages, thus reducing the amount of iron available to the cell (163). Furthermore, as iron levels have also been shown to significantly reduce the efficacy of the anti-TB antibiotics isoniazid and ethambutol, the use of iron chelators may restore the effectiveness of such antibiotics (164). Targeting iron metabolism has the potential to directly inhibit the growth of *Mtb*, by interfering with *Mtb*-specific iron pathways and its survival mechanisms. Additionally, restricting iron availability in host immune cells may also serve to fight *Mtb* infection by stabilizing HIF1α to enhance important inflammatory and metabolic processes central to eradicating the infection. Therefore, we hypothesize that therapeutic iron chelation will function as a double-edged sword by boosting host immunometabolism via the stabilization of HIF1α and by directly starving *Mtb* of iron.

## Fine-tuning HIF1α and iron; a mechanism to support innate host cell function during *Mtb* infection?

Iron chelation may be utilized to artificially trigger HIF1α-mediated pro-inflammatory and glycolytic pathways in host immune cells during *Mtb* infection. In normoxia, HIF1α is usually undetectable due to the inhibitory action of the PHD proteins (165). PHD proteins act by hydroxylating the oxygen-dependent degradation (ODD) domain on HIF1α (166). To function optimally, PHD proteins require oxygen, 2-oxoglutarate, ascorbate and Fe^2+^ to successfully modify the ODD domain on HIF1α (126). When Fe^2+^ levels are low, the activity of the PHD proteins is reduced (167). This is in contrast to hypoxic conditions, where the lack of oxygen inhibits the PHD proteins, thus stabilizing HIF1α (126). This allows heterodimerization of HIF1α with its β-subunit, and translocation into the nucleus where HIF1α binds to hypoxia-response elements linked with a variety of genes involved in various cellular processes, including inducing *pfkfb3* and *IL1*β transcription (168, 169). Moreover, GLUT1, as well as a number of other glycolytic enzymes such as phosphofructokinase, are upregulated to promote anaerobic glycolysis, to compensate for the lack of OXPHOS (170–172). Iron chelator-induced inhibition of PHD proteins and the resulting HIF1α stabilization encompasses the potential to trigger this molecular cascade during *Mtb* infection under aerobic conditions, thus, boosting the pro-inflammatory response of the infected host macrophage and promoting clearance of the infection. Indeed, several studies have shown that HIF is stabilized upon iron chelation in various cell types, including human renal Hep3B cells, human breast cancer MDA468 cells, and hMDMs (173–177). If iron chelation could induce such pro-inflammatory and pro-glycolytic effects during *Mtb* infection, then one may expect the opposite to occur with the addition of iron itself. Indeed, iron has been shown to promote intracellular and extracellular growth of *Mtb* H37Rv in J774A.1 macrophages (178). Moreover, addition of iron significantly reduces TNFα, IL1α, IL1β, and IL6 transcripts, along with TNFα protein levels, during *Mtb* infection (178). Hence, this work demonstrates that the modulation of iron metabolism can potentially regulate the functional relationship between the infected host cell and *Mtb*. Additionally, DFX has been shown to boost the autophagic process, to promote eradication of *Mtb* (179, 180). Western blot and immunofluorescence analyses of murine BMDMs incubated with the iron chelators deferiprone or desferasirox have also been shown to reduce the intracellular growth of *Chlamydia psittaci* and *Legionella pneumophilia* further suggesting that iron chelation may be therapeutically beneficial in the context of *Mtb* infection (181). Even though host-directed iron chelation may bring about reductions in intracellular iron levels, stabilize HIF1α, and trigger pro-inflammatory and glycolytic responses, intrinsic homeostatic mechanisms are still in place to correct for low iron levels thereby limiting host cell stress and toxicity. For example, ferritin, a key intracellular iron storage protein, helps to maintain optimal cellular function upon iron deprivation (154). Indeed, extensive flow cytometry analysis, extracellular metabolic flux analysis and mass spectrometry analysis show that complete ferritin deficiency in myeloid cells dysregulates host energy metabolism and increases susceptibility to *Mtb* H37Rv infection (131). Furthermore, the use of iron chelators have been shown to have no effect, and even reduce, the production of superoxide in *Mtb* H37Rv–infected U937 macrophage cells and THP-1 monocytes while simultaneously reducing the number and viability of *Mtb* mycobacteria (182, 183). Harnessing the potential of PHD proteins and their interconnectivity with HIF1α, through the use of iron chelators, may hold future promise for the development of HDTs for the treatment of TB infection and other infectious diseases. It must also be acknowledged that prolonged induction of HIF1α may cause detrimental damage to lung tissue by promoting excessive inflammation and oxidative stress. For example, in hMDMs and human respiratory A549 cells, HIF1α enhances the expression and secretion of matrix metalloprotease-1 (MMP-1), the main protease implicated in the uncontrolled destruction of lung tissue in TB (184). In fact, HIF1α, which is expressed highly in lung biopsies from patients with pulmonary TB, is necessary for MMP-1 gene expression and secretion (184). Moreover, HIF1α, and DFX, has been shown to positively regulate transcript levels of heme oxygenase−1 (HO-1), an oxidative stress response protein that catalyzes the degradation of heme to Fe^2+^ and other intermediaries (185, 186). HO-1 expression is also markedly increased in rabbits, mice, and non-human primates during experimental *Mtb* Erdman and *Mtb* H37Rv infection and its expression gradually decreases during subsequent successful therapy (187). Moreover, systemic levels of HO-1 are dramatically increased in individuals with active pulmonary and extra-pulmonary tuberculosis (188). Therefore, a thorough understanding of the underlying molecular mechanisms governed by HIF1α during *Mtb* infection would undoubtedly help fine-tune the development of combinatorial host-directed therapeutic approaches, while helping to reduce damage caused to the lung, thus preventing further TB dissemination. Another advantage of stabilizing HIF1α through the use of iron chelators is the potential ability to also boost host immune cell function through the modulation of alternative metabolic pathways such as the PPP, fatty acid metabolism and amino acid metabolism.

## The potential effect of iron, iron chelators and HIF1α stabilization on alternative metabolic pathways

Alternative metabolic pathways are also crucial for cellular growth and function particularly during *Mtb* infection. These alternative metabolic pathways may play a crucial role in the host response to infection and could potentially be targeted by iron chelation therapy. As HIF1α is a well-documented regulator of glycolysis, it is plausible that it can potentially regulate specific alternative metabolic pathways and support immunity during *Mtb* infection. These metabolic pathways include the PPP, fatty acid metabolism and the metabolism of important amino acids, namely glutamine and tryptophan.

The PPP is tightly coupled to glycolysis through the glycolytic intermediate glucose-6-phosphate (G6P), which can be shunted to the PPP to generate NADPH, ribose-5-phospate and other biosynthetic intermediates also utilized in the glycolytic process (189). Coupled with the fact that glycolysis has been shown to be induced upon *Mtb* infection in human AMs, this may also be indicative of an upregulation of the PPP during *Mtb* infection (7). Indeed, the lungs of *Mtb*-infected mice exhibit upregulated gene expression of enzymes involved in both glycolysis and the PPP (83). In this study, transcript levels of the PPP genes *Gpi1, G6pdx*, and *Pgd* were analyzed (83). The first enzyme of the oxidative phase of the PPP, glucose-6-phosphate dehydrogenase (G6PD), is induced by HIF1α in several different cancer cell lines (171, 190–192). Moreover, metabolomic analyses show that HIF1α overexpression results in increased levels of PPP metabolites in murine BMDMs (26). Importantly, the PPP is a major source of NADPH, which is necessary for the production of free radicals like NO and superoxide, and for protecting the cells against oxidative stress (193). The importance of the PPP for ROS production during *Mtb* infection has also been suggested by one study linking G6PD deficiency in humans with increased susceptibility to BCG infections due to impaired ROS production by neutrophils and monocytes (194). Another study investigating metabolomic profiles in murine macrophages treated with iron show increased levels of NADPH and 6-phosphogluconic acid, indicating the potential involvement of other factors, in addition to HIF1α, in the iron-mediated regulation of the PPP (195). Since the upregulation of glycolysis for rapid ATP production is an important host response against *Mtb*, downregulation of the PPP could further increase flux through the glycolytic pathway thus supporting host immune cells further. Whether these observations reflect the findings in iron chelated-*Mtb*-infected host cells has yet to be examined and needs to be investigated.

HIF1α has also been shown to be involved in fatty acid metabolism. Research shows that upon infection with *Mtb*, host cells differentiate into lipid forming foamy macrophages due to pathogen-induced dysregulations in lipid metabolism (113, 115). In an ESAT-6 mediated feedback mechanism, another study shows that *Mtb* actively manipulates host cells into metabolizing fatty acids, by diverting glycolytic metabolism toward ketone body synthesis, by enabling feedback activation of the anti-lipolytic G protein-coupled receptor GPR109A resulting in lipid body accumulation (115). Studies in cancer cells also show that hypoxia boosts the expression of fatty acid synthase and lipin-1 through HIF1α and the sterol regulatory element binding protein resulting in elevated fatty acid synthesis and lipid storage (196, 197). In accordance with that, murine peritoneal macrophages exposed to hypoxia show increased accumulation of lipid droplets, fatty acid synthesis and TAG synthesis (198). Importantly, hypoxic conditions also result in the downregulation of acyl-CoA synthase and acyl-CoA dehydrogenase, two key enzymes of the fatty acid β-oxidation pathway (199). These metabolic conditions could favor growth of mycobacteria, which use host derived fatty acids as a major carbon source (107). However, it remains to be investigated if iron chelation, and subsequent stabilization of HIF1α affects fatty acid metabolism in *Mtb*-infected host cells.

Glutamine metabolism represents another important metabolic pathway during *Mtb* infection. Glutamine metabolism is also a metabolic target of HIF1α signaling. During *Mtb* H37Rv infection, nuclear magnetic resonance analysis of infected C57BL/6 murine lungs shows an upregulation of succinate, which can be generated from glutamine through glutaminolysis (85). Silencing of PHD2 in skeletal cells, which stabilizes HIF1α, results in an increase in glutamine uptake and an increase in the expression of glutaminase-1, the enzyme that catalyzes the conversion of glutamine to glutamate. Glutamate can then be fed into the TCA cycle to produce αKG and succinate. Importantly, glutamate-derived αKG may also be used by the TCA cycle to produce succinate. Increased succinate oxidation by the succinate dehydrogenase (SDH) enhances the production of mitochondrial ROS, which in turn boosts HIF1α and IL1β levels in LPS-stimulated murine BMDMs (102). Furthermore, metabolizing glutamate through the arginosuccinate shunt, which links the TCA cycle with the urea cycle, results in the production of NO (40). Thus, increases in glutamine metabolism may potentially support anti-microbial immune responses in *Mtb*-infected host cells. However, glutamine can be preferentially used for glutathione production rather than being shunted into the TCA cycle (200). Glutathione is known to be an important antioxidant and reducing agent protecting cells from being damaged by oxidizing conditions thus may be critical during *Mtb* infection to protect the delicate lung tissue (201). Whether HIF1α stabilization through iron chelation has similar effects on glutamine metabolism in *Mtb*-infected host cells remains to be seen.

Tryptophan metabolism is another important metabolic pathway regulated through iron and HIF1α. Tryptophan is a crucial amino acid for intracellular bacterial growth and depletion of tryptophan through activation of the kynurenine pathway has been shown to inhibit growth of *Toxoplasma gondii* and *Legionella pneumophila* in monocytes and fibroblasts (202). Mycobacterial growth, however, is unaffected by tryptophan starvation in murine peritoneal macrophages, due to the bacteria's capacity to synthesize tryptophan *de novo* (97). However, picolinic acid, a natural degradation product of tryptophan, inhibits intra-macrophagic growth of *M.avium* and *Mtb in vitro* (93, 202). Nevertheless, IDO1, the first rate-limiting enzyme in kynurenine metabolism, is upregulated in murine BMDMs upon infection with *M.avium*, however, its deficiency does not impact on the outcome of the infection (93). Increased IDO1 activity is known to suppress the protective immune response in rhesus macaques, particularly the production of IFNγ by CD4^+^ T cells, and correlates with a higher bacterial *Mtb* CDC1551burden (203). Therefore, inhibition of IDO1 may be beneficial for TB, in the context of persistent live bacterial infection. Interestingly, IDO1 is a heme-containing enzyme; iron chelation reduces its activity and iron supplementation increases its activity thus the effect of iron chelation on tryptophan metabolism in *Mtb*-infected cells may be promising and warrants further investigation (204).

## Conclusion

**Figure 2:**
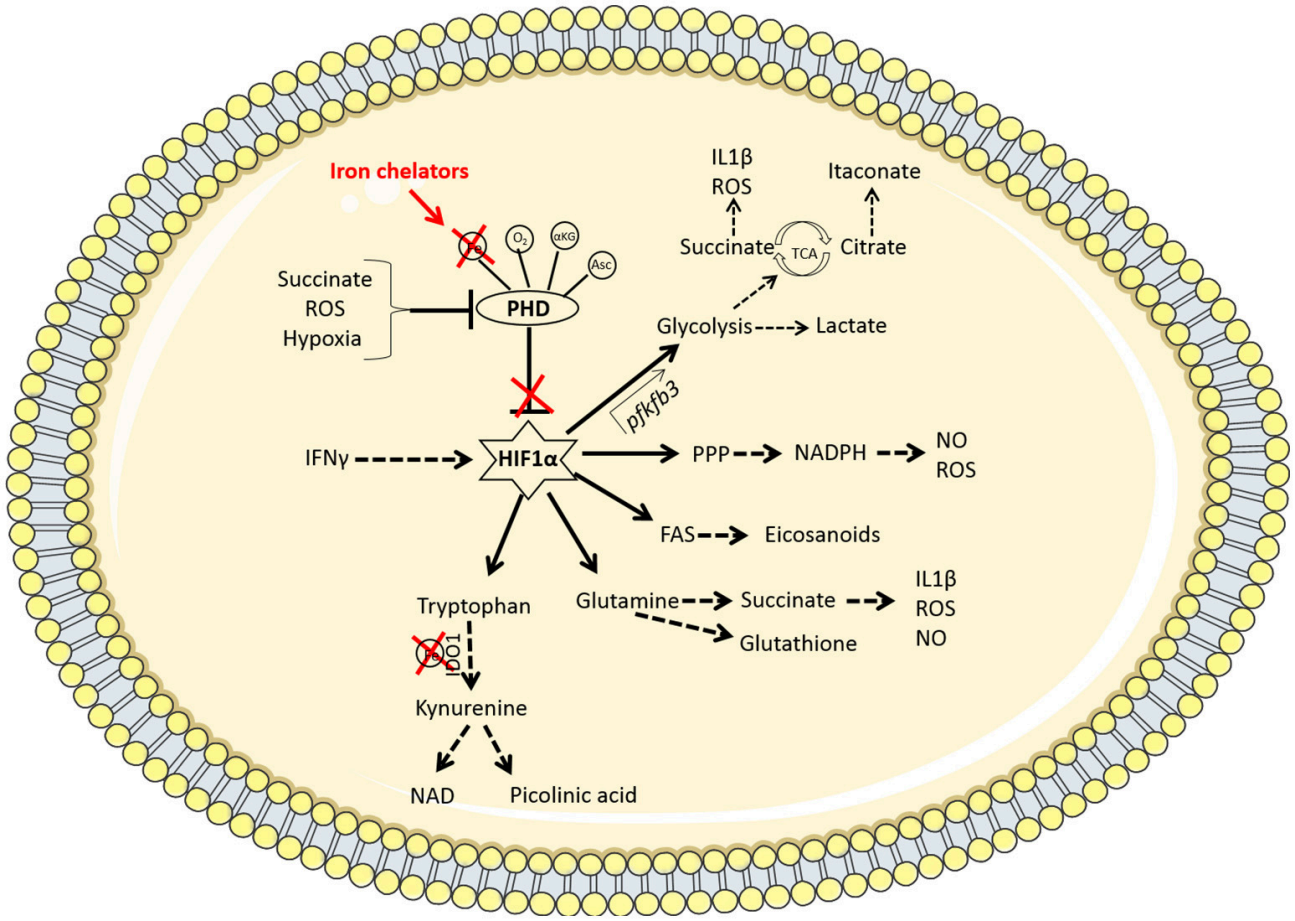
Iron chelators modulate multiple immunometabolic pathways via the stabilization of HIF1α. Under homeostatic conditions, the PHD enzymes hydroxylate HIF1α marking it for degradation. These enzymes require oxygen (O_2_), iron (Fe), α-ketoglutarate (αKG), and ascorbate (Asc) as cofactors to function. When oxygen is low (hypoxia), the PHD enzymes are disabled, resulting in the stabilization of hypoxia-inducible factor 1α (HIF1α). Other factors such as the accumulation of the metabolite succinate or reactive oxygen species (ROS) can also inhibit PHD enzymes. The therapeutic use of iron chelators will reduce the availability of iron inside the cell and therefore deny the PHD enzymes of the iron they require to function. Iron chelators thereby stabilize HIF1α which promotes enhanced flux through glycolysis by binding to the promoter region of the *pfkfb3* gene. This increased rate of glycolysis produces lactate and synthesizes the required building blocks for cellular proliferation and effector function. PAMP or DAMP signals in the macrophage (such as LPS stimulation or infection with *Mtb*, for example) leads to increased aerobic glycolysis and a break in the TCA cycle at 2 points; succinate (which promotes IL1β and ROS production as well as further inhibiting PHD enzymes) and citrate (which leads to the accumulation of the anti-bacterial metabolite, itaconate, via the enzyme IRG-1). HIF1α also mediates increased flux through the pentose phosphate pathway (PPP) which provides NADPH required from NO and ROS production. HIF1α promotes fatty acid synthesis (FAS), leading to the accumulation of lipid droplets and the production of eicosanoids. There is also a role for HIF1α in promoting amino acid metabolism. Glutamine can be used to produce succinate or the antioxidant glutathione. Tryptophan is processed by the iron-dependent enzyme IDO1, which results in a net anti-inflammatory response but can also produce NAD or picolinic acid, which has anti-microbial properties. Thus iron chelation may be a useful tool for manipulating macrophage metabolism during *Mtb* infection through the stabilization of HIF1α.

Despite various treatment options available to treat active TB, the prevalence of drug-resistant TB is increasing, further highlighting the need for novel therapies to fight the bacteria. The majority of individuals infected with *Mtb* mount an adequate innate immune response which results in early clearance of the bacteria. This suggests that supporting myeloid cell function could serve as a host directed preventative or therapeutic strategy. We hypothesize that restricting iron availability, through the use of iron chelators, may be an effective host-directed approach to supporting protective *Mtb*-infected macrophage responses which may enhance early clearance of the infection. As Figure 2 depicts, by depriving macrophages of iron and stabilizing HIF1α, this could potentially function as a double-edged sword by boosting host immunometabolism and by directly starving *Mtb* of iron. In addition to boosting multiple metabolic pathways, HIF1α can directly and indirectly support many key cellular mediators, such as the multi-functional effects of IFNγ. Thus future studies need to investigate the use of iron chelators and their potential to be utilized as a HDT to boost the host immune response to *Mtb* infection.

## Author contributions

JP conceptualized, planned, wrote, proof read, and edited all drafts of the manuscript. SB and JK wrote, planned, proof read and edited all drafts of the manuscript. ST wrote and proof read the manuscript. SM and JS wrote the manuscript.

### Conflict of interest statement

The authors declare that the research was conducted in the absence of any commercial or financial relationships that could be construed as a potential conflict of interest.

## References

[1] Houben RM Dodd PJ. The global burden of latent tuberculosis infection: a re-estimation using mathematical modelling. PLoS Med. (2016) 13:e1002152. 10.1371/journal.pmed.1002152 27780211 PMC5079585

[2] WHO Global Tuberculosis Report 2017. WHO (2017).

[3] Pai M Behr MA Dowdy D Dheda K Divangahi M Boehme CC Tuberculosis. Nat Rev Dis Primers (2016) 2:16076 10.1038/nrdp.2016.76 27784885

[4] Lillebaek T Dirksen A Baess I Strunge B Thomsen VO Andersen AB. Molecular evidence of endogenous reactivation of *Mycobacterium tuberculosis* after 33 years of latent infection. J Infect Dis. (2002) 185:401–4. 10.1086/338342 11807725

[5] Vynnycky E Fine PE. Lifetime risks, incubation period, and serial interval of tuberculosis. Am J Epidemiol. (2000) 152:247–63. 10.1093/aje/152.3.247 10933272

[6] Srivastava S Pasipanodya JG Meek C Leff R Gumbo T Multidrug-resistant tuberculosis not due to noncompliance but to between-patient pharmacokinetic variability. J Infect Dis. (2011) 204:1951–9. 10.1093/infdis/jir658 22021624 PMC3209814

[7] Gleeson LE Sheedy FJ Palsson-McDermott EM Triglia D O'Leary SM O'Sullivan MP. Cutting edge: *Mycobacterium tuberculosis* induces aerobic glycolysis in human alveolar macrophages that is required for control of intracellular bacillary replication. J Immunol. (2016) 196:2444–9. 10.4049/jimmunol.1501612 26873991

[8] Braverman J Sogi KM Benjamin D Nomura DK Stanley SA. HIF-1α is an essential mediator of IFN-γ-dependent immunity to *Mycobacterium tuberculosis*. J Immunol. (2016) 197:1287–97. 10.4049/jimmunol.1600266 27430718 PMC4976004

[9] Koster S Upadhyay S Chandra P Papavinasasundaram K Yang G Hassan A. *Mycobacterium tuberculosis* is protected from NADPH oxidase and LC3-associated phagocytosis by the LCP protein CpsA. Proc Natl Acad Sci USA. (2017) 114:E8711–e20. 10.1073/pnas.1707792114 28973896 PMC5642705

[10] Eileen A Wong CK Keith A Reimann Flynn JL The role of IL-10 during early M. tuberculosis infection in a non-human primate model. J Immunol. (2017) 198:123.5.

[11] Huang ML Becker EM Whitnall M Suryo Rahmanto Y Ponka P Richardson DR. Elucidation of the mechanism of mitochondrial iron loading in Friedreich's ataxia by analysis of a mouse mutant. Proc Natl Acad Sci USA. (2009) 106:16381–6. 10.1073/pnas.0906784106 19805308 PMC2752539

[12] Oexle H Gnaiger E Weiss G. Iron-dependent changes in cellular energy metabolism: influence on citric acid cycle and oxidative phosphorylation. Biochim Biophys Acta (1999) 1413:99–107. 10.1016/S0005-2728(99)00088-2 10556622

[13] Whitnall M Suryo Rahmanto Y Huang ML Saletta F Lok HC Gutierrez L. Identification of nonferritin mitochondrial iron deposits in a mouse model of Friedreich ataxia. Proc Natl Acad Sci USA. (2012) 109:20590–5. 10.1073/pnas.1215349109 23169664 PMC3528580

[14] Meiser J Kramer L Sapcariu SC Battello N Ghelfi J D'Herouel AF. Pro-inflammatory macrophages sustain pyruvate oxidation through pyruvate dehydrogenase for the synthesis of itaconate and to enable cytokine expression. J Biol Chem. (2016) 291:3932–46. 10.1074/jbc.M115.676817 26679997 PMC4759172

[15] Gardiner CM Finlay DK. What fuels natural killers? metabolism and NK cell responses. Front Immunol. (2017) 8:367. 10.3389/fimmu.2017.00367 28421073 PMC5376555

[16] O'Neill LA Kishton RJ Rathmell J. A guide to immunometabolism for immunologists. Nature reviews Immunology. (2016) 16:553–65. 10.1038/nri.2016.70 27396447 PMC5001910

[17] Pearce EJ Everts B. Dendritic cell metabolism. Nat Rev Immunol. (2015) 15:18–29. 10.1038/nri3771 25534620 PMC4495583

[18] Pearce EL Pearce EJ. Metabolic pathways in immune cell activation and quiescence. Immunity (2013) 38:633–43. 10.1016/j.immuni.2013.04.005 23601682 PMC3654249

[19] Poznanski SM Barra NG Ashkar AA Schertzer JD. Immunometabolism of T cells and NK cells: metabolic control of effector and regulatory function. Inflamm Res. (2018) 67:813–28. 10.1007/s00011-018-1174-3 30066126

[20] Van den Bossche J O'Neill LA Menon D Macrophage Immunometabolism: where are we (going)? Trends Immunol. (2017) 38:395–406. 10.1016/j.it.2017.03.001 28396078

[21] Michalek RD Gerriets VA Jacobs SR Macintyre AN MacIver NJ Mason EF. Cutting edge: distinct glycolytic and lipid oxidative metabolic programs are essential for effector and regulatory CD4+ T cell subsets. J Immunol. (2011) 186:3299–303. 10.4049/jimmunol.1003613T 21317389 PMC3198034

[22] Warburg O Wind F Negelein E. The metabolism of tumors in the body. J Gen Physiol. (1927) 8:519–30. 10.1085/jgp.8.6.519 19872213 PMC2140820

[23] Gubser PM Bantug GR Razik L Fischer M Dimeloe S Hoenger G. Rapid effector function of memory CD8+ T cells requires an immediate-early glycolytic switch. Nat Immunol. (2013) 14:1064–72. 10.1038/ni.2687 23955661

[24] Keating SE Zaiatz-Bittencourt V Loftus RM Keane C Brennan K Finlay DK. Metabolic reprogramming supports IFN-gamma production by CD56bright NK cells. J Immunol. (2016) 196:2552–60. 10.4049/jimmunol.1501783 26873994

[25] Everts B Amiel E Huang SC Smith AM Chang CH Lam WY. TLR-driven early glycolytic reprogramming via the kinases TBK1-IKKvarepsilon supports the anabolic demands of dendritic cell activation. Nat Immunol. (2014) 15:323–32. 10.1038/ni.2833 24562310 PMC4358322

[26] Wang T Liu H Lian G Zhang SY Wang X Jiang C. HIF1α-induced glycolysis metabolism is essential to the activation of inflammatory macrophages. Mediators Inflamm. (2017) 2017:9029327. 10.1155/2017/9029327 29386753 PMC5745720

[27] Lawless SJ Kedia-Mehta N Walls JF McGarrigle R Convery O Sinclair LV. Glucose represses dendritic cell-induced T cell responses. Nat Commun. (2017) 8:15620. 10.1038/ncomms15620 28555668 PMC5459989

[28] Amiel E Everts B Fritz D Beauchamp S Ge B Pearce EL. Mechanistic target of rapamycin inhibition extends cellular lifespan in dendritic cells by preserving mitochondrial function. J Immunol. (2014) 193:2821–30. 10.4049/jimmunol.1302498 25108022 PMC4302759

[29] Tan Z Xie N Cui H Moellering DR Abraham E Thannickal VJ. Pyruvate dehydrogenase kinase 1 participates in macrophage polarization via regulating glucose metabolism. J Immunol. (2015) 194:6082–9. 10.4049/jimmunol.1402469 25964487 PMC4458459

[30] Gleeson LE Ryan D O'Leary SM McLaughlin AM Sheedy FJ Keane JM. Cigarette smoking impairs the bioenergetic immune response to Mycobacterium tuberculosis infection. Am J Resp Cell Mol Biol. (2018). [Epub ahead of print]. 10.1165/rcmb.2018-0162OC 29944387

[31] Cheng SC Quintin J Cramer RA Shepardson KM Saeed S Kumar V. mTOR- and HIF-1alpha-mediated aerobic glycolysis as metabolic basis for trained immunity. Science (2014) 345:1250684. 10.1126/science.1250684 25258083 PMC4226238

[32] Arts RJW Carvalho A La Rocca C Palma C Rodrigues F Silvestre R. Immunometabolic pathways in BCG-induced trained immunity. Cell Rep. (2016) 17:2562–71. 10.1016/j.celrep.2016.11.011 27926861 PMC5177620

[33] Castano D Barrera LF Rojas M. *Mycobacterium tuberculosis* alters the differentiation of monocytes into macrophages *in vitro*. Cell Immunol. (2011) 268:60–7. 10.1016/j.cellimm.2011.02.006 21420074

[34] Giacomini E Iona E Ferroni L Miettinen M Fattorini L Orefici G. Infection of human macrophages and dendritic cells with *Mycobacterium tuberculosis* induces a differential cytokine gene expression that modulates T cell response. J Immunol. (2001) 166:7033–41. 10.4049/jimmunol.166.12.7033 11390447

[35] Bystrom J Evans I Newson J Stables M Toor I van Rooijen N. Resolution-phase macrophages possess a unique inflammatory phenotype that is controlled by cAMP. Blood (2008) 112:4117–27. 10.1182/blood-2007-12-129767 18779392 PMC2581990

[36] Huang Z Luo Q Guo Y Chen J Xiong G Peng Y. *Mycobacterium tuberculosis*-induced polarization of human macrophage orchestrates the formation and development of tuberculous granulomas *in vitro*. PloS ONE (2015) 10:e0129744. 10.1371/journal.pone.0129744 26091535 PMC4474964

[37] Marino S Cilfone NA Mattila JT Linderman JJ Flynn JL Kirschner DE. Macrophage polarization drives granuloma outcome during *Mycobacterium tuberculosis* infection. Infect Immun. (2015) 83:324–38. 10.1128/IAI.02494-14 25368116 PMC4288886

[38] West AP Brodsky IE Rahner C Woo DK Erdjument-Bromage H Tempst P. TLR signalling augments macrophage bactericidal activity through mitochondrial ROS. Nature. (2011) 472(7344):476-80. 10.1038/nature09973 21525932 PMC3460538

[39] Tannahill GM Curtis AM Adamik J Palsson-McDermott EM McGettrick AF Goel G. Succinate is an inflammatory signal that induces IL-1beta through HIF-1alpha. Nature 496:238–42. 10.1038/nature11986 23535595 PMC4031686

[40] Jha AK Huang SC Sergushichev A Lampropoulou V Ivanova Y Loginicheva E. Network integration of parallel metabolic and transcriptional data reveals metabolic modules that regulate macrophage polarization. Immunity (2015) 42:419–30. 10.1016/j.immuni.2015.02.005 25786174

[41] Donnelly S Stack CM O'Neill SM Sayed AA Williams DL Dalton JP. Helminth 2-Cys peroxiredoxin drives Th2 responses through a mechanism involving alternatively activated macrophages. FASEB J. (2008) 22:4022–32. 10.1096/fj.08-106278 18708590 PMC3980656

[42] Vats D Mukundan L Odegaard JI Zhang L Smith KL Morel CR. Oxidative metabolism and PGC-1beta attenuate macrophage-mediated inflammation. Cell Metabol. (2006) 4:13–24. 10.1016/j.cmet.2006.05.011 16814729 PMC1904486

[43] McKinney JD Honer zu Bentrup K Munoz-Elias EJ Miczak A Chen B Chan WT. Persistence of *Mycobacterium tuberculosis* in macrophages and mice requires the glyoxylate shunt enzyme isocitrate lyase. Nature (2000) 406:735–8. 10.1038/35021074 10963599

[44] Mills EL Ryan DG Prag HA Dikovskaya D Menon D Zaslona Z. Itaconate is an anti-inflammatory metabolite that activates Nrf2 via alkylation of KEAP1. Nature (2018) 556:113–7. 10.1038/nature25986 29590092 PMC6047741

[45] Michelucci A Cordes T Ghelfi J Pailot A Reiling N Goldmann O. Immune-responsive gene 1 protein links metabolism to immunity by catalyzing itaconic acid production. Proc Natl Acad Sci USA. (2013) 110:7820–5. 10.1073/pnas.1218599110 23610393 PMC3651434

[46] D'Avila H Melo RC Parreira GG Werneck-Barroso E Castro-Faria-Neto HC Bozza PT. *Mycobacterium bovis* bacillus Calmette-Guerin induces TLR2-mediated formation of lipid bodies: intracellular domains for eicosanoid synthesis *in vivo*. J Immunol. (2006) 176:3087–97. 10.4049/jimmunol.176.5.3087 16493068

[47] Pacheco P Bozza FA Gomes RN Bozza M Weller PF Castro-Faria-Neto HC. Lipopolysaccharide-induced leukocyte lipid body formation *in vivo:* innate immunity elicited intracellular loci involved in eicosanoid metabolism. J Immunol. (2002) 169:6498–506. 10.4049/jimmunol.169.11.6498 12444160

[48] Huang YL Morales-Rosado J Ray J Myers TG Kho T Lu M. Toll-like receptor agonists promote prolonged triglyceride storage in macrophages. J Biol Chem. (2014) 289:3001–12. 10.1074/jbc.M113.524587t 24337578 PMC3908431

[49] Rahaman SO Lennon DJ Febbraio M Podrez EA Hazen SL Silverstein RL. A CD36-dependent signaling cascade is necessary for macrophage foam cell formation. Cell Metabol. (2006) 4:211–21. 10.1016/j.cmet.2006.06.007 16950138 PMC1855263

[50] Singh V Kaur C Chaudhary VK Rao KV Chatterjee S M. Tuberculosis Secretory Protein ESAT-6 Induces metabolic flux perturbations to drive foamy macrophage differentiation. Sci Rep. (2015) 5:12906 10.1038/srep12906 26250836 PMC5388048

[51] Lachmandas E Boutens L Ratter JM Hijmans A Hooiveld GJ Joosten LA. Microbial stimulation of different Toll-like receptor signalling pathways induces diverse metabolic programmes in human monocytes. Nat Microbiol. (2016) 2:16246. 10.1038/nmicrobiol.2016.246. 27991883

[52] Rodriguez-Espinosa O Rojas-Espinosa O Moreno-Altamirano MM Lopez-Villegas EO Sanchez-Garcia FJ. Metabolic requirements for neutrophil extracellular traps formation. Immunology (2015) 145:213–24. 10.1111/imm.12437 25545227 PMC4427386

[53] Sbarra AJ Karnovsky ML. The biochemical basis of phagocytosis. I. Metabolic changes during the ingestion of particles by polymorphonuclear leukocytes. J Biol Chem. (1959) 234:1355–62. 13654378

[54] Azevedo EP Rochael NC Guimaraes-Costa AB de Souza-Vieira TS Ganilho J Saraiva EM. A metabolic shift toward pentose phosphate pathway is necessary for amyloid fibril- and phorbol 12-myristate 13-acetate-induced Neutrophil Extracellular Trap (NET) formation. J Biol Chem. (2015) 290:22174–83. 10.1074/jbc.M115.640094 26198639 PMC4571968

[55] Furukawa S Saito H Inoue T Matsuda T Fukatsu K Han I. Supplemental glutamine augments phagocytosis and reactive oxygen intermediate production by neutrophils and monocytes from postoperative patients *in vitro*. Nutrition (2000) 16:323–9. 10.1016/S0899-9007(00)00228-8 10793298

[56] Kirchner T Moller S Klinger M Solbach W Laskay T Behnen M. The impact of various reactive oxygen species on the formation of neutrophil extracellular traps. Mediators Inflamm. (2012) 2012:849136. 10.1155/2012/849136 22481865 PMC3317033

[57] Cramer T Yamanishi Y Clausen BE Forster I Pawlinski R Mackman N. HIF-1alpha is essential for myeloid cell-mediated inflammation. Cell (2003) 112:645–57. 10.1016/S0092-8674(03)00154-5 12628185 PMC4480774

[58] Donnelly RP Loftus RM Keating SE Liou KT Biron CA Gardiner CM. mTORC1-dependent metabolic reprogramming is a prerequisite for NK cell effector function. J Immunol. (2014) 193:4477–84. 10.4049/jimmunol.1401558 25261477 PMC4201970

[59] Keppel MP Saucier N Mah AY Vogel TP Cooper MA. Activation-specific metabolic requirements for NK Cell IFN-gamma production. J Immunol. (2015) 194:1954–62. 10.4049/jimmunol.1402099 25595780 PMC4323953

[60] Viel S Marcais A Guimaraes FS Loftus R Rabilloud J Grau M. TGF-beta inhibits the activation and functions of NK cells by repressing the mTOR pathway. Sci Signal. (2016) 9:ra19. 10.1126/scisignal.aad1884 26884601

[61] Brill KJ Li Q Larkin R Canaday DH Kaplan DR Boom WH. Human natural killer cells mediate killing of intracellular *Mycobacterium tuberculosis* H37Rv via granule-independent mechanisms. Infect Immun. (2001) 69:1755–65. 10.1128/IAI.69.3.1755-1765.2001 11179353 PMC98082

[62] Vankayalapati R Klucar P Wizel B Weis SE Samten B Safi H. NK cells regulate CD8+ T cell effector function in response to an intracellular pathogen. J Immunol. (2004) 172:130–7. 10.4049/jimmunol.172.1.130 14688318

[63] Fu X Liu Y Li L Li Q Qiao D Wang H. Human natural killer cells expressing the memory-associated marker CD45RO from tuberculous pleurisy respond more strongly and rapidly than CD45RO- natural killer cells following stimulation with interleukin-12. Immunology (2011) 134:41–9. 10.1111/j.1365-2567.2011.03464.x 21711347 PMC3173693

[64] Fu X Yu S Yang B Lao S Li B Wu C Memory-Like Antigen-Specific Human NK Cells from TB Pleural Fluids Produced IL-22 in Response to IL-15 or *Mycobacterium tuberculosis* Antigens. PloS ONE (2016) 11:e0151721 10.1371/journal.pone.0151721 27031950 PMC4816314

[65] Krawczyk CM Holowka T Sun J Blagih J Amiel E DeBerardinis RJ. Toll-like receptor-induced changes in glycolytic metabolism regulate dendritic cell activation. Blood (2010) 115:4742–9. 10.1182/blood-2009-10-249540 20351312 PMC2890190

[66] Everts B Amiel E van der Windt GJ Freitas TC Chott R Yarasheski KE. Commitment to glycolysis sustains survival of NO-producing inflammatory dendritic cells. Blood (2012) 120:1422–31. 10.1182/blood-2012-03-419747 22786879 PMC3423780

[67] Jantsch J Chakravortty D Turza N Prechtel AT Buchholz B Gerlach RG. Hypoxia and hypoxia-inducible factor-1 alpha modulate lipopolysaccharide-induced dendritic cell activation and function. J Immunol. (2008) 180:4697–705. 10.4049/jimmunol.180.7.4697 18354193

[68] Lozza L Farinacci M Bechtle M Staber M Zedler U Baiocchini A. Communication between human dendritic cell subsets in tuberculosis: requirements for naive CD4(+) T cell stimulation. Front Immunol (2014) 5:324. 10.3389/fimmu.2014.00324 25071784 PMC4094910

[69] Wang R Dillon CP Shi LZ Milasta S Carter R Finkelstein D. The transcription factor Myc controls metabolic reprogramming upon T lymphocyte activation. Immunity (2011) 35:871–82. 10.1016/j.immuni.2011.09.021 22195744 PMC3248798

[70] Chen W Jin W Hardegen N Lei KJ Li L Marinos N. Conversion of peripheral CD4+CD25- naive T cells to CD4+CD25+ regulatory T cells by TGF-beta induction of transcription factor Foxp3. J Exp Med. (2003) 198:1875–86. 10.1084/jem.20030152 14676299 PMC2194145

[71] Davidson TS DiPaolo RJ Andersson J Shevach EM. Cutting Edge: IL-2 is essential for TGF-beta-mediated induction of Foxp3+ T regulatory cells. J Immunol. (2007) 178:4022–6. 10.4049/jimmunol.178.7.4022 17371955

[72] Hsieh CS Macatonia SE Tripp CS Wolf SF O'Garra A Murphy KM. Development of TH1 CD4+ T cells through IL-12 produced by Listeria-induced macrophages. Science (1993) 260:547–9. 10.1126/science.8097338 8097338

[73] Korn T Bettelli E Gao W Awasthi A Jager A Strom TB. IL-21 initiates an alternative pathway to induce proinflammatory T(H)17 cells. Nature (2007) 448:484–7. 10.1038/nature05970 17581588 PMC3805028

[74] Le Gros G Ben-Sasson SZ Seder R Finkelman FD Paul WE Generation of interleukin 4 (IL-4)-producing cells *in vivo* and *in vitro*: IL-2 and IL-4 are required for *in vitro* generation of IL-4-producing cells. J Exp Med. (1990) 172:921–9. 10.1084/jem.172.3.921 2117636 PMC2188542

[75] Lighvani AA Frucht DM Jankovic D Yamane H Aliberti J Hissong BD. T-bet is rapidly induced by interferon-gamma in lymphoid and myeloid cells. Proc Natl Acad Sci USA. (2001) 98:15137–42. 10.1073/pnas.261570598 11752460 PMC64996

[76] Swain SL Weinberg AD English M Huston G. IL-4 directs the development of Th2-like helper effectors. J Immunol. (1990) 145:3796–806. 2147202

[77] Shi LZ Wang R Huang G Vogel P Neale G Green DR. HIF1alpha-dependent glycolytic pathway orchestrates a metabolic checkpoint for the differentiation of TH17 and Treg cells. J Exp Med. (2011) 208:1367–76. 10.1084/jem.20110278 21708926 PMC3135370

[78] van der Windt GJ O'Sullivan D Everts B Huang SC Buck MD Curtis JD CD8 memory T cells have a bioenergetic advantage that underlies their rapid recall ability. Proc Natl Acad Sci USA. (2013) 110:14336–41. 10.1073/pnas.1221740110 23940348 PMC3761631

[79] O'Sullivan D van der Windt GJ Huang SC Curtis JD Chang CH Buck MD. Memory CD8(+) T cells use cell-intrinsic lipolysis to support the metabolic programming necessary for development. Immunity (2014) 41:75–88. 10.1016/j.immuni.2014.06.005 25001241 PMC4120664

[80] Strauss L Czystowska M Szajnik M Mandapathil M Whiteside TL. Differential responses of human regulatory T cells (Treg) and effector T cells to rapamycin. PloS ONE (2009) 4:e5994. 10.1371/journal.pone.0005994 19543393 PMC2694984

[81] Das A Ranganathan V Umar D Thukral S George A Rath S. Effector/memory CD4 T cells making either Th1 or Th2 cytokines commonly co-express T-bet and GATA-3. PloS ONE (2017) 12:e0185932. 10.1371/journal.pone.0185932 29088218 PMC5663332

[82] Hirota K Duarte JH Veldhoen M Hornsby E Li Y Cua DJ. Fate mapping of IL-17-producing T cells in inflammatory responses. Nat Immunol. (2011) 12:255–63. 10.1038/ni.1993 21278737 PMC3040235

[83] Shi L Salamon H Eugenin EA Pine R Cooper A Gennaro ML. Infection with *Mycobacterium tuberculosis* induces the Warburg effect in mouse lungs. Sci Rep. (2015) 5:18176. 10.1038/srep18176 26658723 PMC4674750

[84] Kaplan G Post FA Moreira AL Wainwright H Kreiswirth BN Tanverdi M. *Mycobacterium tuberculosis* growth at the cavity surface: a microenvironment with failed immunity. Infect Immun. (2003) 71:7099–108. 10.1128/IAI.71.12.7099-7108.2003 14638800 PMC308931

[85] Shin JH Yang JY Jeon BY Yoon YJ Cho SN Kang YH. (1)H NMR-based metabolomic profiling in mice infected with *Mycobacterium tuberculosis*. J Proteome Res. (2011) 10:2238–47. 10.1021/pr101054m 21452902

[86] Huang L Nazarova EV Tan S Liu Y Russell DG. Growth of *Mycobacterium tuberculosis in vivo* segregates with host macrophage metabolism and ontogeny. J Exp Med. (2018) 215:1135–52. 10.1084/jem.20172020 29500179 PMC5881470

[87] Aston C Rom WN Talbot AT Reibman J Early inhibition of mycobacterial growth by human alveolar macrophages is not due to nitric oxide. Am J Resp Crit Care Med. (1998) 157(6 Pt 1):1943–50. 10.1164/ajrccm.157.6.9705028 9620931

[88] Schenk M Fabri M Krutzik SR Lee DJ Vu DM Sieling PA. Interleukin-1beta triggers the differentiation of macrophages with enhanced capacity to present mycobacterial antigen to T cells. Immunology (2014) 141:174–80. 10.1111/imm.12167 24032597 PMC3904238

[89] Duque-Correa MA Kuhl AA Rodriguez PC Zedler U Schommer-Leitner S Rao M. Macrophage arginase-1 controls bacterial growth and pathology in hypoxic tuberculosis granulomas. Proc Natl Acad Sci USA. (2014) 111:E4024–32. 10.1073/pnas.1408839111 25201986 PMC4183271

[90] Pessanha AP Martins RA Mattos-Guaraldi AL Vianna A Moreira LO. Arginase-1 expression in granulomas of tuberculosis patients. FEMS Immunol Med Microbiol. (2012) 66:265–8. 10.1111/j.1574-695X.2012.01012.x 22827286

[91] Suzuki Y Miwa S Akamatsu T Suzuki M Fujie M Nakamura Y. Indoleamine 2,3-dioxygenase in the pathogenesis of tuberculous pleurisy. Int J Tuberc Lung Dis. (2013) 17:1501–6. 10.5588/ijtld.13.0082 24125458

[92] Suzuki Y Suda T Asada K Miwa S Suzuki M Fujie M. Serum indoleamine 2,3-dioxygenase activity predicts prognosis of pulmonary tuberculosis. Clin Vaccine Immunol. (2012) 19:436–42. 10.1128/CVI.05402-11 22219312 PMC3294601

[93] Blumenthal A Nagalingam G Huch JH Walker L Guillemin GJ Smythe GA. M. tuberculosis induces potent activation of IDO-1, but this is not essential for the immunological control of infection. PloS ONE (2012) 7:e37314. 10.1371/journal.pone.0037314 22649518 PMC3359358

[94] Cowley S Ko M Pick N Chow R Downing KJ Gordhan BG. The *Mycobacterium tuberculosis* protein serine/threonine kinase PknG is linked to cellular glutamate/glutamine levels and is important for growth *in vivo*. Mol Microbiol. (2004) 52:1691–702. 10.1111/j.1365-2958.2004.04085.x 15186418

[95] Mattila JT Ojo OO Kepka-Lenhart D Marino S Kim JH Eum SY. Microenvironments in tuberculous granulomas are delineated by distinct populations of macrophage subsets and expression of nitric oxide synthase and arginase isoforms. J Immunol. (2013) 191:773–84. 10.4049/jimmunol.1300113 23749634 PMC3746594

[96] Tullius MV Harth G Horwitz MA. Glutamine synthetase GlnA1 is essential for growth of *Mycobacterium tuberculosis* in human THP-1 macrophages and guinea pigs. Infect Immun. (2003) 71:3927–36. 10.1128/IAI.71.7.3927-3936.2003 12819079 PMC162033

[97] Zhang YJ Reddy MC Ioerger TR Rothchild AC Dartois V Schuster BM. Tryptophan biosynthesis protects mycobacteria from CD4 T-cell-mediated killing. Cell (2013) 155:1296–308. 10.1016/j.cell.2013.10.045 24315099 PMC3902092

[98] Fallarino F Grohmann U You S McGrath BC Cavener DR Vacca C. The combined effects of tryptophan starvation and tryptophan catabolites down-regulate T cell receptor zeta-chain and induce a regulatory phenotype in naive T cells. J Immunol. (2006) 176:6752–61. 10.4049/jimmunol.176.11.6752 16709834

[99] Popov A Abdullah Z Wickenhauser C Saric T Driesen J Hanisch FG. Indoleamine 2,3-dioxygenase-expressing dendritic cells form suppurative granulomas following Listeria monocytogenes infection. J Clin Invest. (2006) 116:3160–70. 10.1172/JCI28996 17111046 PMC1636691

[100] van Laarhoven A Dian S Aguirre-Gamboa R Avila-Pacheco J Ricano-Ponce I Ruesen C Cerebral tryptophan metabolism and outcome of tuberculous meningitis: an observational cohort study. Lancet Infect Dis. (2018) 18:526–35. 10.1016/S1473-3099(18)30053-7 29395996

[101] Gordon AH Hart PD Young MR. Ammonia inhibits phagosome-lysosome fusion in macrophages. Nature (1980) 286:79–80. 10.1038/286079a0 6993961

[102] Mills EL Kelly B Logan A Costa ASH Varma M Bryant CE. Succinate dehydrogenase supports metabolic repurposing of mitochondria to drive inflammatory macrophages. Cell (2016) 167:457–70 e13. 10.1016/j.cell.2016.08.064 27667687 PMC5863951

[103] Dodd CE Pyle CJ Glowinski R Rajaram MVS Schlesinger LS. CD36-mediated uptake of surfactant lipids by human macrophages promotes intracellular growth of *Mycobacterium tuberculosis*. J Immunol. (2016) 197:4727–35. 10.4049/jimmunol.1600856 27913648 PMC5137803

[104] Singh A Crossman DK Mai D Guidry L Voskuil MI Renfrow MB. *Mycobacterium tuberculosis* WhiB3 maintains redox homeostasis by regulating virulence lipid anabolism to modulate macrophage response. PLoS Pathog. (2009) 5:e1000545. 10.1371/journal.ppat.1000545 19680450 PMC2718811

[105] Baek SH Li AH Sassetti CM. Metabolic regulation of mycobacterial growth and antibiotic sensitivity. PLoS Biol. (2011) 9:e1001065. 10.1371/journal.pbio.1001065 21629732 PMC3101192

[106] Cole ST Brosch R Parkhill J Garnier T Churcher C Harris D. Deciphering the biology of *Mycobacterium tuberculosis* from the complete genome sequence. Nature (1998) 393:537–44. 9634230 10.1038/31159

[107] Lee W VanderVen BC Fahey RJ Russell DG. Intracellular *Mycobacterium tuberculosis* exploits host-derived fatty acids to limit metabolic stress. J Biol Chem. (2013) 288:6788–800. 10.1074/jbc.M112.445056 23306194 PMC3591590

[108] Podinovskaia M Lee W Caldwell S Russell DG. Infection of macrophages with *Mycobacterium tuberculosis* induces global modifications to phagosomal function. Cell Microbiol. (2013) 15:843–59. 10.1111/cmi.12092 23253353 PMC3620910

[109] Rajaram MV Brooks MN Morris JD Torrelles JB Azad AK Schlesinger LS. *Mycobacterium tuberculosis* activates human macrophage peroxisome proliferator-activated receptor gamma linking mannose receptor recognition to regulation of immune responses. J Immunol. (2010) 185:929–42. 10.4049/jimmunol.1000866 20554962 PMC3014549

[110] Olivier M Tanck MW Out R Villard EF Lammers B Bouchareychas L. Human ATP-binding cassette G1 controls macrophage lipoprotein lipase bioavailability and promotes foam cell formation. Arterioscler Thromb Vasc Biol. (2012) 32:2223–31. 10.1161/ATVBAHA.111.243519 22772754

[111] Munoz-Elias EJ McKinney JD. *Mycobacterium tuberculosis* isocitrate lyases 1 and 2 are jointly required for *in vivo* growth and virulence. Nat Med. (2005) 11:638–44. 10.1038/nm1252 15895072 PMC1464426

[112] Pandey AK Sassetti CM. Mycobacterial persistence requires the utilization of host cholesterol. Proc Natl Acad Sci USA. (2008) 105:4376–80. 10.1073/pnas.0711159105 18334639 PMC2393810

[113] Peyron P Vaubourgeix J Poquet Y Levillain F Botanch C Bardou F. Foamy macrophages from tuberculous patients' granulomas constitute a nutrient-rich reservoir for M. tuberculosis persistence. PLoS Pathog. (2008) 4:e1000204. 10.1371/journal.ppat.1000204 19002241 PMC2575403

[114] Jain M Petzold CJ Schelle MW Leavell MD Mougous JD Bertozzi CR. Lipidomics reveals control of *Mycobacterium tuberculosis* virulence lipids via metabolic coupling. Proc Natl Acad Sci USA. (2007) 104:5133–8. 10.1073/pnas.0610634104 17360366 PMC1829275

[115] Singh V Jamwal S Jain R Verma P Gokhale R Rao KV. *Mycobacterium tuberculosis*-driven targeted recalibration of macrophage lipid homeostasis promotes the foamy phenotype. Cell Host Microbe (2012) 12:669–81. 10.1016/j.chom.2012.09.012 23159056

[116] Knight M Braverman J Asfaha K Gronert K Stanley S. Lipid droplet formation in *Mycobacterium tuberculosis* infected macrophages requires IFN-gamma/HIF-1alpha signaling and supports host defense. PLoS Pathog. (2018) 14:e1006874. 10.1371/journal.ppat.1006874 29370315 PMC5800697

[117] Phelan JJ O'Hanlon C Reynolds JV O'Sullivan J The role of energy metabolism in driving disease progression in inflammatory, hypoxic and angiogenic microenvironments. Gastro Open J. (2015) 1:44–58. 10.17140/GOJ-1-108.

[118] Phelan JJ Feighery R Eldin OS Meachair SO Cannon A Byrne R. Examining the connectivity between different cellular processes in the Barrett tissue microenvironment. Cancer Lett. (2016) 371:334–46. 10.1016/j.canlet.2015.11.041 26688097

[119] Braverman J Stanley SA. Nitric Oxide Modulates macrophage responses to *Mycobacterium tuberculosis* infection through activation of HIF-1alpha and repression of NF-kappaB. J Immunol. (2017) 199:1805–16. 10.4049/jimmunol.1700515 28754681 PMC5568107

[120] Obach M Navarro-Sabate A Caro J Kong X Duran J Gomez M. 6-Phosphofructo-2-kinase (pfkfb3) gene promoter contains hypoxia-inducible factor-1 binding sites necessary for transactivation in response to hypoxia. J Biol Chem. (2004) 279:53562–70. 10.1074/jbc.M406096200 15466858

[121] Chesney J Mitchell R Benigni F Bacher M Spiegel L Al-Abed Y. An inducible gene product for 6-phosphofructo-2-kinase with an AU-rich instability element: role in tumor cell glycolysis and the Warburg effect. Proc Natl Acad Sci USA. (1999) 96:3047–52. 10.1073/pnas.96.6.3047 10077634 PMC15892

[122] Subbian S Tsenova L Kim MJ Wainwright HC Visser A Bandyopadhyay N. Lesion-specific immune response in granulomas of patients with pulmonary tuberculosis: a pilot study. PloS ONE (2015) 10:e0132249. 10.1371/journal.pone.0132249 26133981 PMC4489805

[123] Subbian S Tsenova L Yang G O'Brien P Parsons S Peixoto B. Chronic pulmonary cavitary tuberculosis in rabbits: a failed host immune response. Open Biol. (2011) 1:110016. 10.1098/rsob.110016 22645653 PMC3352086

[124] Flynn JL Chan J Triebold KJ Dalton DK Stewart TA Bloom BR. An essential role for interferon gamma in resistance to *Mycobacterium tuberculosis* infection. J Exp Med. (1993) 178:2249–54. 10.1084/jem.178.6.2249 7504064 PMC2191274

[125] Marxsen JH Stengel P Doege K Heikkinen P Jokilehto T Wagner T. Hypoxia-inducible factor-1 (HIF-1) promotes its degradation by induction of HIF-alpha-prolyl-4-hydroxylases. Biochem J. (2004) 381(Pt 3):761–7. 10.1042/BJ20040620 15104534 PMC1133886

[126] Bruick RK McKnight SL. A conserved family of prolyl-4-hydroxylases that modify HIF. Science (2001) 294:1337–40. 10.1126/science.1066373 11598268

[127] Siegert I Schödel J Nairz M Schatz V Dettmer K Dick C. Ferritin-Mediated Iron sequestration stabilizes hypoxia-inducible factor-1α upon lps activation in the presence of ample oxygen. Cell Rep. (2015) 13:2048–55. 10.1016/j.celrep.2015.11.005 26628374

[128] McKie AT Barrow D Latunde-Dada GO Rolfs A Sager G Mudaly E. An iron-regulated ferric reductase associated with the absorption of dietary iron. Science (2001) 291:1755–9. 10.1126/science.1057206 11230685

[129] Taha DA Thanoon IA. Antioxidant status, C-reactive protein and iron status in patients with pulmonary tuberculosis. Sultan Qaboos Univ Med J. (2010) 10:361–9. 21509257 PMC3074724

[130] Gangadhar T Srikanth P Suneetha Y Predictive value of iron store markers in anemia of chronic kidney disease. J Chem Pharm Res. (2010) 2:400–10.

[131] Reddy VP Chinta KC Saini V Glasgow JN Hull TD Traylor A. Ferritin H deficiency in myeloid compartments dysregulates host energy metabolism and increases susceptibility to mycobacterium tuberculosis infection. Front Immunol. (2018) 9:860. 10.3389/fimmu.2018.00860 29774023 PMC5943674

[132] McKie AT Marciani P Rolfs A Brennan K Wehr K Barrow D. A novel duodenal iron-regulated transporter, IREG1, implicated in the basolateral transfer of iron to the circulation. Mol Cell (2000) 5:299–309. 10.1016/S1097-2765(00)80425-6 10882071

[133] Brown PJ Johnson PM. Isolation of a transferrin receptor structure from sodium deoxycholate-solubilized human placental syncytiotrophoblast plasma membrane. Placenta (1981) 2:1–10. 6259638

[134] Rotig A de Lonlay P Chretien D Foury F Koenig M Sidi D. Aconitase and mitochondrial iron-sulphur protein deficiency in Friedreich ataxia. Nat Gene. (1997) 17:215–7. 10.1038/ng1097-215 9326946

[135] Muhlenhoff U Richhardt N Gerber J Lill R. Characterization of iron-sulfur protein assembly in isolated mitochondria. A requirement for ATP, NADH, and reduced iron. J Biol Chem. (2002) 277:29810–6. 10.1074/jbc.M204675200t 12065597

[136] Galy B Ferring-Appel D Becker C Gretz N Grone HJ Schumann K. Iron regulatory proteins control a mucosal block to intestinal iron absorption. Cell Rep. (2013) 3:844–57. 10.1016/j.celrep.2013.02.026 23523353

[137] Nemeth E Tuttle MS Powelson J Vaughn MB Donovan A Ward DM. Hepcidin regulates cellular iron efflux by binding to ferroportin and inducing its internalization. Science (2004) 306:2090–3. 10.1126/science.1104742 15514116

[138] Pietrangelo A. Genetics, genetic testing, and management of hemochromatosis: 15 years since hepcidin. Gastroenterology (2015) 149:1240–51.e4. 10.1053/j.gastro.2015.06.045 26164493

[139] Poggiali E Cassinerio E Zanaboni L Cappellini MD. An update on iron chelation therapy. Blood Transfus. (2012) 10:411–22. 10.2450/2012.0008-12 22790257 PMC3496216

[140] Silva B Faustino P. An overview of molecular basis of iron metabolism regulation and the associated pathologies. Biochim Biophys Acta. (2015) 1852:1347–59. 10.1016/j.bbadis.2015.03.011 25843914

[141] Cloonan SM Glass K Laucho-Contreras ME Bhashyam AR Cervo M Pabon MA. Mitochondrial iron chelation ameliorates cigarette smoke-induced bronchitis and emphysema in mice. Nat Med. (2016) 22:163–74. 10.1038/nm.4021 26752519 PMC4742374

[142] Smith HJ Meremikwu M Iron chelating agents for treating malaria. Cochrane Database Syst Rev. (2003) CD001474. 10.1002/14651858.CD001474 12804409

[143] Georgiou NA van der Bruggen T Oudshoorn M Nottet HS Marx JJ van Asbeck BS. Inhibition of human immunodeficiency virus type 1 replication in human mononuclear blood cells by the iron chelators deferoxamine, deferiprone, and bleomycin. J Infect Dis. (2000) 181:484–90. 10.1086/315223 10669330

[144] Thompson MG Corey BW Si Y Craft DW Zurawski DV. Antibacterial activities of iron chelators against common nosocomial pathogens. Antimicrob Agents Chem. (2012) 56:5419–21. 10.1128/AAC.01197-12 22850524 PMC3457357

[145] Kim CM Shin SH. Effect of iron-chelator deferiprone on the *in vitro* growth of *staphylococci*. J Korean Medi Sci. (2009) 24:289–95. 10.3346/jkms.2009.24.2.289 19399272 PMC2672130

[146] Gobin J Horwitz MA. Exochelins of *Mycobacterium tuberculosis* remove iron from human iron-binding proteins and donate iron to mycobactins in the M. tuberculosis cell wall. J Exp Med. (1996) 183:1527–32. 8666910 10.1084/jem.183.4.1527 PMC2192514

[147] Pandey R Rodriguez GM. IdeR is required for iron homeostasis and virulence in *Mycobacterium tuberculosis*. Mol Microbiol. (2014) 91:98–109. 10.1111/mmi.12441 24205844 PMC3902104

[148] Gordeuk VR McLaren CE MacPhail AP Deichsel G Bothwell TH. Associations of iron overload in Africa with hepatocellular carcinoma and tuberculosis: Strachan's 1929 thesis revisited. Blood (1996) 87:3470–6. 8605366

[149] Gangaidzo IT Moyo VM Mvundura E Aggrey G Murphree NL Khumalo H. Association of pulmonary tuberculosis with increased dietary iron. J Infect Dis. (2001) 184:936–9. 10.1086/323203 11528590

[150] Boelaert JR Weinberg GA Weinberg ED. Altered iron metabolism in HIV infection: mechanisms, possible consequences, and proposals for management. Infect Agents Dis. (1996) 5:36–46. 8789598

[151] Thompson AB Bohling T Heires A Linder J Rennard SI. Lower respiratory tract iron burden is increased in association with cigarette smoking. J Lab Clin Med. (1991) 117:493–9. 2045717

[152] Kurthkoti K Amin H Marakalala MJ Ghanny S Subbian S Sakatos A. The capacity of mycobacterium tuberculosis to survive iron starvation might enable it to persist in iron-deprived microenvironments of human granulomas. MBio (2017) 8:e01092–17. 10.1128/mBio.01092-17 28811344 PMC5559634

[153] Agoro R Mura C. Inflammation-induced up-regulation of hepcidin and down-regulation of ferroportin transcription are dependent on macrophage polarization. Blood Cells Mol Dis. (2016) 61:16–25. 10.1016/j.bcmd.2016.07.006 27667162

[154] Abreu R Quinn F Giri PK. Role of the hepcidin-ferroportin axis in pathogen-mediated intracellular iron sequestration in human phagocytic cells. Blood Adv. (2018) 2:1089–100. 10.1182/bloodadvances.2017015255 29764842 PMC5965048

[155] Abreu R Essler L Loy A Quinn F Giri P. Heparin inhibits intracellular *Mycobacterium tuberculosis* bacterial replication by reducing iron levels in human macrophages. Sci Rep. (2018) 8:7296. 10.1038/s41598-018-25480-y 29740038 PMC5940867

[156] Gomes MS Appelberg R. NRAMP1- or cytokine-induced bacteriostasis of Mycobacterium avium by mouse macrophages is independent of the respiratory burst. Microbiology (2002) 148(Pt 10):3155–60. 10.1099/00221287-148-10-3155t 12368449

[157] Zwilling BS Kuhn DE Wikoff L Brown D Lafuse W. Role of iron in Nramp1-mediated inhibition of mycobacterial growth. Infect Immun. (1999) 67:1386–92. 10024586 10.1128/iai.67.3.1386-1392.1999 PMC96472

[158] Bellamy R Ruwende C Corrah T McAdam KP Whittle HC Hill AV. Variations in the NRAMP1 gene and susceptibility to tuberculosis in West Africans. N Engl J Med. (1998) 338:640–4. 10.1056/NEJM199803053381002 9486992

[159] Ryu S Park YK Bai GH Kim SJ Park SN Kang S. 3′UTR polymorphisms in the NRAMP1 gene are associated with susceptibility to tuberculosis in Koreans. Int J Tuberc Lung Dis. (2000) 4:577–80. 10864190

[160] An YC Feng FM Yuan JX Ji CM Wang YH Guo M. [Study on the association of INT4 and 3'UTR polymorphism of natural-resistance-associated macrophage protein 1 gene with susceptibility to pulmonary tuberculosis]. Zhonghua liu xing bing xue za zhi (2006) 27:37–40. 16737570

[161] Gomes MS Appelberg R. Evidence for a link between iron metabolism and Nramp1 gene function in innate resistance against *Mycobacterium avium*. Immunology (1998) 95:165–8. 10.1046/j.1365-2567.1998.00630.x 9824471 PMC1364300

[162] Coleman MM Basdeo SA Coleman AM Ni Cheallaigh C Peral de Castro C McLaughlin AM. All-trans retinoic acid augments autophagy during intracellular bacterial infection. Am J Resp Cell Mol Biol. (2018) [Epub ahead of print]. 10.1165/rcmb.2017-0382OC 29852080

[163] Iturralde M Vass JK Oria R Brock JH. Effect of iron and retinoic acid on the control of transferrin receptor and ferritin in the human promonocytic cell line U937. Biochim Biophys Acta (1992) 1133:241–6. 10.1016/0167-4889(92)90043-B 1737056

[164] Lounis N Maslo C Truffot-Pernot C Grosset J Boelaert RJ. Impact of iron loading on the activity of isoniazid or ethambutol in the treatment of murine tuberculosis. Int J Tuberc Lung Dis. (2003) 7:575–9. 12797701

[165] Huang LE Gu J Schau M Bunn HF. Regulation of hypoxia-inducible factor 1alpha is mediated by an O2-dependent degradation domain via the ubiquitin-proteasome pathway. Proc Natil Acad Sci USA. (1998) 95:7987–92. 10.1073/pnas.95.14.7987 9653127 PMC20916

[166] Masson N Willam C Maxwell PH Pugh CW Ratcliffe PJ. Independent function of two destruction domains in hypoxia-inducible factor-alpha chains activated by prolyl hydroxylation. EMBO J. (2001) 20:5197–206. 10.1093/emboj/20.18.5197 11566883 PMC125617

[167] Nandal A Ruiz JC Subramanian P Ghimire-Rijal S Sinnamon RA Stemmler TL. Activation of the HIF prolyl hydroxylase by the iron chaperones PCBP1 and PCBP2. Cell Metabol. (2011) 14:647–57. 10.1016/j.cmet.2011.08.015 22055506 PMC3361910

[168] Frede S Freitag P Otto T Heilmaier C Fandrey J. The proinflammatory cytokine interleukin 1beta and hypoxia cooperatively induce the expression of adrenomedullin in ovarian carcinoma cells through hypoxia inducible factor 1 activation. Cancer Res. (2005) 65:4690–7. 10.1158/0008-5472.CAN-04-3877 15930287

[169] Minchenko A Leshchinsky I Opentanova I Sang N Srinivas V Armstead V. Hypoxia-inducible factor-1-mediated expression of the 6-phosphofructo-2-kinase/fructose-2,6-bisphosphatase-3 (PFKFB3) gene. Its possible role in the Warburg effect. J Biol Chem. (2002) 277:6183–7. 10.1074/jbc.M110978200 11744734 PMC4518871

[170] Del Rey MJ Valin A Usategui A Garcia-Herrero CM Sanchez-Arago M Cuezva JM Hif-1alpha knockdown reduces glycolytic metabolism and induces cell death of human synovial fibroblasts under normoxic conditions. Sci Rep. (2017) 7:3644 10.1038/s41598-017-03921-4 28623342 PMC5473902

[171] Ebert BL Firth JD Ratcliffe PJ. Hypoxia and mitochondrial inhibitors regulate expression of glucose transporter-1 via distinct Cis-acting sequences. J Biol Chem. (1995) 270:29083–9. 10.1074/jbc.270.49.29083 7493931

[172] Frezza C Zheng L Tennant DA Papkovsky DB Hedley BA Kalna G. Metabolic profiling of hypoxic cells revealed a catabolic signature required for cell survival. PloS ONE (2011) 6:e24411. 10.1371/journal.pone.0024411 21912692 PMC3166325

[173] Wang GL Semenza GL. Desferrioxamine induces erythropoietin gene expression and hypoxia-inducible factor 1 DNA-binding activity: implications for models of hypoxia signal transduction. Blood (1993) 82:3610–5. 8260699

[174] Kir D Saluja M Modi S Venkatachalam A Schnettler E Roy S. Cell-permeable iron inhibits vascular endothelial growth factor receptor-2 signaling and tumor angiogenesis. Oncotarget (2016) 7:65348–63. 10.18632/oncotarget.11689 27589831 PMC5323160

[175] Bartolome S Dhillon NK Buch S Casillan AJ Wood JG O'Brien-Ladner AR. Deferoxamine mimics the pattern of hypoxia-related injury at the microvasculature. Shock (2009) 31:481–5. 10.1097/SHK.0b013e318188db14 18827748

[176] Chong TW Horwitz LD Moore JW Sowter HM Harris AL. A mycobacterial iron chelator, desferri-exochelin, induces hypoxia-inducible factors 1 and 2, NIP3, and vascular endothelial growth factor in cancer cell lines. Cancer Res. (2002) 62:6924–7. 12460908

[177] Tian YM Yeoh KK Lee MK Eriksson T Kessler BM Kramer HB. Differential sensitivity of hypoxia inducible factor hydroxylation sites to hypoxia and hydroxylase inhibitors. J Biol Chem. (2011) 286:13041–51. 10.1074/jbc.M110.211110 21335549 PMC3075650

[178] Serafin-Lopez J Chacon-Salinas R Munoz-Cruz S Enciso-Moreno JA Estrada-Parra SA Estrada-Garcia I. The effect of iron on the expression of cytokines in macrophages infected with *Mycobacterium tuberculosis*. Scand J Immunol. (2004) 60:329–37. 10.1111/j.0300-9475.2004.01482.x 15379857

[179] Ivana De Domenico DMW Kushner JP Kaplan J Iron chelation by deferoxamine induces autophagy. Blood (2008) 112:117.

[180] Gutierrez MG Master SS Singh SB Taylor GA Colombo MI Deretic V. Autophagy is a defense mechanism inhibiting BCG and *Mycobacterium tuberculosis* survival in infected macrophages. Cell (2004) 119:753–66. 10.1016/j.cell.2004.11.038 15607973

[181] Paradkar PN De Domenico I Durchfort N Zohn I Kaplan J Ward DM. Iron depletion limits intracellular bacterial growth in macrophages. Blood (2008) 112:866–74. 10.1182/blood-2007-12-126854 18369153 PMC2481528

[182] Cronje L Edmondson N Eisenach KD Bornman L. Iron and iron chelating agents modulate *Mycobacterium tuberculosis* growth and monocyte-macrophage viability and effector functions. FEMS Immunol Med Microbiol. (2005) 45:103–12. 10.1016/j.femsim.2005.02.007 16051061

[183] Ellis S Kalinowski DS Leotta L Huang ML Jelfs P Sintchenko V. Potent antimycobacterial activity of the pyridoxal isonicotinoyl hydrazone analog 2-pyridylcarboxaldehyde isonicotinoyl hydrazone: a lipophilic transport vehicle for isonicotinic acid hydrazide. Mol Pharmacol. (2014) 85:269–78. 10.1124/mol.113.090357 24243647 PMC6067633

[184] Belton M Brilha S Manavaki R Mauri F Nijran K Hong YT. Hypoxia and tissue destruction in pulmonary TB. Thorax (2016) 71:1145–53. 10.1136/thoraxjnl-2015-207402 27245780 PMC5136721

[185] Basdeo SA Campbell NK Sullivan LM Flood B Creagh EM Mantle TJ. Suppression of human alloreactive T cells by linear tetrapyrroles; relevance for transplantation. Transl Res. (2016) 178:81–94.e2. 10.1016/j.trsl.2016.07.011 27497182

[186] Yang ZZ Zou AP. Transcriptional regulation of heme oxygenases by HIF-1alpha in renal medullary interstitial cells. Am J Physiol Renal Physiol. (2001) 281:F900–8. 10.1152/ajprenal.2001.281.5.F900 11592948

[187] Rockwood N Costa DL Amaral EP Du Bruyn E Kubler A Gil-Santana L. *Mycobacterium tuberculosis* induction of heme oxygenase-1 expression is dependent on oxidative stress and reflects treatment outcomes. Front Immunol. (2017) 8:542. 10.3389/fimmu.2017.00542 28553288 PMC5427075

[188] Andrade BB Pavan Kumar N Mayer-Barber KD Barber DL Sridhar R Rekha VV. Plasma heme oxygenase-1 levels distinguish latent or successfully treated human tuberculosis from active disease. PloS ONE (2013) 8:e62618. 10.1371/journal.pone.0062618 23671613 PMC3646008

[189] Brekke EM Walls AB Schousboe A Waagepetersen HS Sonnewald U Quantitative importance of the pentose phosphate pathway determined by incorporation of 13C from [2-13C]- and [3-13C]glucose into TCA cycle intermediates and neurotransmitter amino acids in functionally intact neurons. J Cereb Blood Flow Metabol (2012) 32:1788–99. 10.1038/jcbfm.2012.85 PMC3434630 22714050

[190] Guo S Miyake M Liu KJ Shi H. Specific inhibition of hypoxia inducible factor 1 exaggerates cell injury induced by *in vitro* ischemia through deteriorating cellular redox environment. J Neurochem. (2009) 108:1309–21. 10.1111/j.1471-4159.2009.05877.x 19183269 PMC2666308

[191] Semenza GL Roth PH Fang HM Wang GL. Transcriptional regulation of genes encoding glycolytic enzymes by hypoxia-inducible factor 1. J Biol Chem. (1994) 269:23757–63. 8089148

[192] Bergeron M Yu AY Solway KE Semenza GL Sharp FR. Induction of hypoxia-inducible factor-1 (HIF-1) and its target genes following focal ischaemia in rat brain. Eur J Neurosci. (1999) 11:4159–70. 10.1046/j.1460-9568.1999.00845.x 10594641

[193] Hothersall JS Gordge M Noronha-Dutra AA. Inhibition of NADPH supply by 6-aminonicotinamide: effect on glutathione, nitric oxide and superoxide in J774 cells. FEBS Lett. (1998) 434:97–100. 10.1016/S0014-5793(98)00959-4 9738459

[194] Khan TA Mazhar H Nawaz M Kalsoom K Ishfaq M Asif H. Expanding the clinical and genetic spectrum of G6PD deficiency: the occurrence of BCGitis and novel missense mutation. Microb Pathog. (2017) 102:160–5. 10.1016/j.micpath.2016.11.025 27914961

[195] Polati R Castagna A Bossi AM Alberio T De Domenico I Kaplan J. Murine macrophages response to iron. J Proteom. (2012) 76 Spec No:10–27. 10.1016/j.jprot.2012.07.018 22835775

[196] Furuta E Pai SK Zhan R Bandyopadhyay S Watabe M Mo YY. Fatty acid synthase gene is up-regulated by hypoxia via activation of Akt and sterol regulatory element binding protein-1. Cancer Res. (2008) 68:1003–11. 10.1158/0008-5472.CAN-07-2489 18281474

[197] Valli A Rodriguez M Moutsianas L Fischer R Fedele V Huang HL. Hypoxia induces a lipogenic cancer cell phenotype via HIF1alpha-dependent and -independent pathways. Oncotarget (2015) 6:1920–41. 10.18632/oncotarget.3058 25605240 PMC4385826

[198] Lu M Kho T Munford RS. Prolonged triglyceride storage in macrophages: pHo trumps pO2 and TLR4. J Immunol. (2014) 193:1392–7. 10.4049/jimmunol.1400886 24973452 PMC4108542

[199] Bostrom P Magnusson B Svensson PA Wiklund O Boren J Carlsson LM. Hypoxia converts human macrophages into triglyceride-loaded foam cells. Arterioscl Thrombo Vascul Biol. (2006) 26:1871–6. 10.1161/01.ATV.0000229665.78997.0b 16741148

[200] Stegen S van Gastel N Eelen G Ghesquiere B D'Anna F Thienpont B. HIF-1alpha promotes glutamine-mediated redox homeostasis and glycogen-dependent bioenergetics to support postimplantation bone cell survival. Cell Metabol. (2016) 23:265–79. 10.1016/j.cmet.2016.01.002 26863487 PMC7611069

[201] Ben-Yoseph O Boxer PA Ross BD. Assessment of the role of the glutathione and pentose phosphate pathways in the protection of primary cerebrocortical cultures from oxidative stress. J Neurochem. (1996) 66:2329–37. 8632155 10.1046/j.1471-4159.1996.66062329.x

[202] Pais TF Appelberg R. Macrophage control of mycobacterial growth induced by picolinic acid is dependent on host cell apoptosis. J Immunol. (2000) 164:389–97. 10.4049/jimmunol.164.1.389 10605034

[203] Gautam US Foreman TW Bucsan AN Veatch AV Alvarez X Adekambi T. *In vivo* inhibition of tryptophan catabolism reorganizes the tuberculoma and augments immune-mediated control of *Mycobacterium tuberculosis*. Proc Natl Acad Sci USA. (2018) 115:E62–71. 10.1073/pnas.1711373114 29255022 PMC5776797

[204] Thomas SR Salahifar H Mashima R Hunt NH Richardson DR Stocker R. Antioxidants inhibit indoleamine 2,3-dioxygenase in IFN-gamma-activated human macrophages: posttranslational regulation by pyrrolidine dithiocarbamate. J Immunol. (2001) 166:6332–40. 10.4049/jimmunol.166.10.6332 11342657

